# MicroRNAs and Alarmins in Cardio-Oncology: Biomarkers and Therapeutic Implications in Skin and Breast Cancer

**DOI:** 10.3390/ijms27188329

**Published:** 2026-09-19

**Authors:** Federica Cannistrà, Sara Sileno, Francesco Cribari, Marco D’Agostino, Francesco Martino, Francesco Morrone, Guido Melillo, Federica Limana, Alessandra Magenta

**Affiliations:** 1 Institute of Translational Pharmacology (IFT), National Research Council of Italy (CNR), 00133 Rome, Italy; federicacannistra1999@gmail.com; 2 Laboratory of Molecular Regenerative Medicine, Istituto Dermopatico dell’Immacolata, IDI-IRCCS, 00167 Rome, Italy; sara.silenoo@gmail.com (S.S.); marcodagostino86@hotmail.it (M.D.); 3 Unit of Cardiology, Istituto Dermopatico dell’Immacolata, IDI-IRCCS, 00167 Rome, Italy; frarib93@gmail.com (F.C.); g.melillo@idi.it (G.M.); 4 Department of Internal Medicine, Anaesthesiology and Cardiovascular Sciences, Sapienza University of Rome, 00161 Rome, Italy; francesco.martino@uniroma1.it; 5 Complex Operating Unit of Paediatrics and Neonatal Care, Spoke Hospital of Corigliano-Rossano, 87064 Cosenza, Italy; f.morrone@aspcs.it; 6 Dipartimento di Promozione delle Scienze Umane e della Qualità della Vita, San Raffaele University of Rome, 00166 Rome, Italy; federica.limana@uniroma5.it; 7 Laboratorio di Patologia Cellulare e Molecolare, IRCCS San Raffaele Roma, 00166 Rome, Italy

**Keywords:** microRNAs, alarmins, cancer therapy, cardiovascular diseases, cardiotoxicity

## Abstract

Cardio-oncology explores the complex interplay between cancer biology and cardiovascular health, particularly the cardiotoxic effects of cancer therapies. Both microRNAs (miRNAs) and alarmins have emerged as key molecular mediators linking tumor progression and cardiac stress, representing promising biomarkers and therapeutic targets. In skin and breast cancer, specific miRNAs, including miR-21, miR-155, miR-34a, and the miR-200 family, regulate tumor proliferation, invasion, metastasis, and therapy resistance. Alarmins such as NPM, HMGB1, HSPs, IL-33, and S100 proteins modulate inflammatory signaling, oxidative stress, and tissue remodeling, contributing to both tumor progression and cardiomyocyte dysfunction. These molecules are also implicated in chemotherapy- and targeted therapy-induced cardiotoxicity, including apoptosis, fibrosis, and impaired cardiac function. This review highlights the dual role of miRNAs and alarmins in tumor biology and cardiovascular toxicity, emphasizing their potential as circulating biomarkers for early detection and monitoring. Therapeutic strategies targeting this axis—including miRNA mimics/inhibitors and alarmin modulators—may provide synergistic benefits by reducing cardiotoxicity while suppressing tumor growth. Understanding miRNA–alarmin relationships, including direct interactions such as the NPM/miR-200c axis, as well as their broader regulatory networks in cardio-oncology, offers novel avenues for precision medicine, enabling integrated approaches to monitor, prevent, and treat therapy-related cardiac complications in patients with skin and breast cancers.

## 1. Introduction

Cardio-oncology is an emerging discipline focused on the cardiovascular health of cancer patients and survivors. Many effective cancer therapies—including traditional chemotherapy, novel targeted agents, immunotherapies, and radiation—can damage the heart, leading to cancer therapy-related cardiotoxicity (CTRCD) (summarized in Table 1). This damage ranges from asymptomatic declines in cardiac function to heart failure, arrhythmias, and other serious cardiovascular events. Preventing or mitigating cardiotoxicity is critical, as it enables patients to receive life-saving cancer treatments without undue risk of heart failure [1,2].

In breast cancer [3] and melanoma [4] (two paradigmatic models in cardio-oncology), cardiovascular care must address cancer therapy-related toxicity across a broad clinical spectrum, including ventricular dysfunction and heart failure, hypertension, arrhythmias and QT prolongation, and vascular complications.

In breast carcinoma, anthracyclines (e.g., doxorubicin, DOX) represent the paradigm of dose-dependent cardiotoxicity: oxidative stress and topoisomerase IIβ-mediated genotoxic damage converge to drive mitochondrial dysfunction and cardiomyocyte loss, thereby promoting adverse remodeling and heart failure (HF). Anti-HER2 therapies, frequently administered sequentially or in combination with chemotherapy, may induce ventricular dysfunction that is more often potentially reversible, consistent with interference with cardioprotective neuregulin–ErbB signaling. Additional drug classes contribute distinct cardiovascular phenotypes: taxanes may exacerbate myocardial dysfunction and increase cumulative cardiac burden in complex regimens; CDK4/6 inhibitors (particularly ribociclib) require careful monitoring for QT prolongation; and inhibitors of the PI3K/AKT/mTOR pathway (such as alpelisib and everolimus) increase cardiometabolic vulnerability through hyperglycemia, dyslipidemia, and edema, thereby amplifying vascular risk [3].

In melanoma, combined BRAF/MEK inhibition is associated with hypertension, left ventricular dysfunction, QT prolongation, and arrhythmias through mechanisms including the suppression of cardioprotective MAPK signaling and contributions from endothelial dysfunction and vascular inflammation [4].

Despite their widespread clinical use, routine surveillance with cardiac troponins and natriuretic peptides in cardio-oncology is supported by relatively limited and heterogeneous evidence. Their interpretability is constrained by several factors, including lack of standardized timing and thresholds, significant inter-assay variability, frequent baseline elevations related to age, renal dysfunction or cancer-related inflammation, and their tendency to rise only after myocardial injury has already occurred. In this context, the 2022 cardio-oncology guidelines emphasize comprehensive baseline cardiovascular assessment and multimodal longitudinal surveillance rather than reliance on isolated biomarker measurements [5].

Within this therapeutic landscape, circulating microRNAs—remarkably stable and deeply integrated into cellular stress and injury networks—emerge as promising candidates for early surveillance and mechanistic phenotyping of cardiotoxicity [4].

microRNAs (miRNAs) have recently gained attention in cardio-oncology as potential tools for early detection, risk stratification, and even therapeutic intervention in therapy-related cardiotoxicity. miRNAs are short (~22-nucleotide) non-coding RNAs that post-transcriptionally regulate gene expression by binding messenger RNAs. They play fundamental roles in both cardiac biology and cancer biology, modulating pathways of cell survival, growth, differentiation, and stress responses. Notably, many miRNAs are tissue-specific (some enriched in the heart or vasculature, others in immune cells or tumors) and are stable in the circulation, making them attractive non-invasive biomarkers. In the heart, miRNAs govern processes like myocardial development, contractility, hypertrophy, and fibrosis, while in cancer, certain “oncomiRs” [6] (oncogenic miRNAs) and tumor-suppressor miRNAs influence tumor growth and metastasis [6]. These small regulators can thus serve as a molecular bridge between oncology and cardiology: cancer therapies can dysregulate cardiac miRNAs (directly or *via* treatment-induced stress), and conversely, manipulating miRNA levels might protect the heart without compromising antitumor efficacy [7].

Alarmins, also known as damage-associated molecular patterns or DAMPs, are endogenous proteins released from dying cells or damaged cells that trigger innate immune system activation [8]. They can cause cardiotoxicity by inducing sterile inflammation *via* Toll-like receptor 4 (TLR4) and RAGE activation, leading to cytokine release and inflammatory cell infiltration. Therefore, they act as mediators in different cardiovascular pathologies, including myocardial ischemia, heart failure, and chemotherapy-induced cardiotoxicity [9,10,11].

This review provides a comprehensive overview of the roles of miRNAs and alarmins in cardio-oncology, focusing on their mechanistic involvement in cardiotoxicity, their utility as early biomarkers across various cancer therapies, and emerging strategies that use miRNA-based therapeutics and/or alarmin inhibition to prevent or mitigate cardiac injury. We integrate insights from the latest (2022) cardio-oncology guidelines [2] and recent literature to highlight current evidence, knowledge gaps, and future potential use of miRNAs and alarmins in cardio-oncology.

Moreover, we provide an example of the close interplay between miRNAs and alarmins, demonstrating that an alarmin can bind and protect miRNAs from RNase-mediated degradation, thereby facilitating their export into the extracellular space and further supporting the interplay between these two classes of molecules.

**Table 1:** Anticancer therapies used in breast and skin cancers and their cardiovascular toxicities.

Cancer Setting	Cancer Treatment (Examples)	Cardiovascular Toxicities (Reported Incidence)
Breast cancer	AC (DOX, epirubicin)	CTRCD/HF (~3-5%); LV dysfunction; supraventricular arrhythmias; ventricular arrhythmias; conduction disorders; myocarditis; pericarditis (rare) [2].
Breast cancer (HER2+)	Anti-HER2 monoclonal antibodies (trastuzumab ± pertuzumab)	CTRCD/LV dysfunction (~8%); HF (~2%) [2].
Breast cancer (advanced, subtype-specific)	ADC (T-DM1, T-DXd, sacituzumab govitecan)	CTRCD/LV dysfunction (~2% with HER2-targeted ADCs); HF; QTc prolongation (~8% with T-DXd); sacituzumab-specific cardiovascular incidence not established [12].
Breast cancer	Taxanes (paclitaxel, docetaxel)	Sinus bradycardia (up to ~30% in early paclitaxel series); AV block; supraventricular arrhythmias; ventricular arrhythmias; myocardial ischemia; coronary vasospasm; hypotension; fluid retention; HF (~2-8%) [2,13].
Breast cancer (HR+)	Endocrine therapy—SERM (tamoxifen)	VTE (~2-3%); QTc prolongation [2].
Breast cancer (HR+, postmenopausal)	Endocrine therapy—aromatase inhibitors (letrozole, anastrozole, exemestane)	Dyslipidemia; metabolic syndrome; hypertension; CAD/angina (~3.9%); MI (~1.1%); HF (~2.1%) [2]
Breast cancer (HR+/HER2−)	CDK4/6 inhibitors (ribociclib, palbociclib, abemaciclib)	QTc prolongation (~5% mainly with ribociclib); ventricular arrhythmias; VTE (~2%) [2]
Breast cancer (selected advanced settings)	PI3K/AKT/mTOR pathway inhibitors (alpelisib, everolimus, capivasertib)	Hyperglycemia (~17-59% agent dependent); dyslipidemia; hypertension; peripheral edema; CTRCD; HF [14,15].
Breast cancer (gBRCA-mutated)	PARP inhibitors (olaparib, talazoparib)	Hypertension (~18%); MACE (~5%); thromboembolitic events (~4%); VTE [2]
Breast cancer (selected regimens)	Fluoropyrimidines (5-fluorouracil, capecitabine)	Coronary vasospasm; angina; myocardial ischemia (~2.2%); overall fluoropyrimidine-associated cardiotoxicity (~5%); ACS; MI; supraventricular arrhythmias; ventricular arrhythmias; myocarditis; pericarditis; Takotsubo syndrome; HF; sudden cardiac death [2]
Breast cancer (selected regimens)	Platinum agents (cisplatin, carboplatin)	Arterial thrombosis (<1% with cisplatin); VTE (~2% with cisplatin); myocardial ischemia; ACS; MI; stroke; hypertension; endothelial dysfunction [2].
Breast cancer (left-sided/chest exposure)	RT	Cardiac events/CAD (~5% cumulative incidence at 10 years); valvular heart disease; pericarditis; pericardial effusion; constrictive pericarditis; cardiomyopathy; HF; conduction disease; arrhythmias; autonomic dysfunction [2]
Melanoma	BRAF inhibitors (vemurafenib, dabrafenib, encorafenib)	MI; AF; sinus bradycardia; supraventricular tachycardia; QTc prolongation; hypertension (~14%); LVEF decline (~2%); pulmonary embolism (<1%) [2]
Melanoma	MEK inhibitors (trametinib, cobimetinib, binimetinib)	Hypertension (~3-15%); LVEF decline (~4-23% trial ranges); HF; pulmonary embolism; sinus bradycardia; peripheral edema [2].
Melanoma	BRAF + MEK combinations (dabrafenib + trametinib; encorafenib + binimetinib; vemurafenib + cobimetinib)	Hypertension (~20%); CTRCD/ LVEF decline (~8%); HF; pulmonary embolism (~2%); VTE; QTc prolongation; MI; AF; sinus bradycardia; supraventricular tachycardia; peripheral edema [2].
Selected TNBC, melanoma and MCC	ICI (anti-PD-1/PD-L1 ± anti-CTLA-4; pembrolizumab, nivolumab, cemiplimab, avelumab, ipilimumab)	Overall CV adverse events (~1%); myocarditis (~0.2–0.3%; ~0.7% with dual ICI); myopericarditis; pericarditis; pericardial effusion; CTRCD; non-inflammatory HF; Takotsubo syndrome; ACS; MI; accelerated atherosclerosis; hypertension; AV block; supraventricular arrhythmias; ventricular arrhythmias; sudden cardiac death; ischemic stroke; VTE; dyslipidemia [2].
Melanoma (advanced)	Anti-LAG-3 + anti-PD-1 (relatlimab + nivolumab)	Myocarditis (~1.7%); myopericarditis; pericarditis; pericardial effusion; CTRCD; HF; Takotsubo syndrome; ACS; MI; AV block; supraventricular arrhythmias; ventricular arrhythmias; sudden cardiac death; ischemic stroke; VTE; dyslipidemia [16].
NMSC (especially cSCC/advanced BCC)	Anti-PD-1 (cemiplimab, pembrolizumab)	Overall PD-1-related CV adverse events (~1%, class-level); myocarditis (~0.2%, class-level); myopericarditis; pericarditis; pericardial effusion; CTRCD; HF; Takotsubo syndrome; ACS; MI; AV block; supraventricular arrhythmias; ventricular arrhythmias; sudden cardiac death; ischemic stroke; VTE; dyslipidemia [2].
Advanced cSCC (selected settings)	EGFR-targeted therapy (cetuximab)	Infusion-related reactions (~8%); hypotension; hypertension; electrolyte disturbances; QTc prolongation; arrhythmias; myocardial ischemia; ACS; HF; cardiovascular-specific event incidence not reliably established [17].
Advanced BCC	Hedgehog pathway inhibitors (vismodegib, sonidegib)	No clinically meaningful QTc prolongation demonstrated in dedicated studies; evidence regarding other cardiovascular toxicities remains limited [18].
Skin cancers (site-dependent)	RT (field-dependent cardiac exposure)	CAD; valvular heart disease; pericarditis; pericardial effusion; constrictive pericarditis; cardiomyopathy; HF; conduction disease; arrhythmias; carotid artery stenosis (>50%; pooled prevalence ~25% after head/neck irradiation); peripheral arterial disease [2].

Abbreviations: AC, anthracycline chemotherapy; ACS, acute coronary syndrome; ADC, antibody-drug conjugate; AF, atrial fibrillation; AV, atrioventricular; BCC, basal cell carcinoma; BRAF, B-Raf proto-oncogene serine/threonine kinase; CAD, coronary artery disease; CDK, cyclin-dependent kinase; cSCC, cutaneous squamous cell carcinoma; CTLA-4, cytotoxic T-lymphocyte-associated protein 4; CTRCD, cancer therapy-related cardiac dysfunction; CV, cardiovascular; DOX, doxorubicin; EGFR, epidermal growth factor receptor; HER2, human epidermal growth factor receptor 2; HF, heart failure; HR, hormone receptor; ICI, immune checkpoint inhibitor; LAG-3, lymphocyte-activation gene 3; LV, left ventricular; LVEF, left ventricular ejection fraction; MACE, major adverse cardiovascular events; MCC, Merkel cell carcinoma; MEK, mitogen-activated protein kinase; MI, myocardial infarction; mTOR, mechanistic target of rapamycin; NMSC, non-melanoma skin cancer; PARP, poly(ADP-ribose) polymerase; PD-1, programmed cell death protein 1; PD-L1, programmed death-ligand 1; PI3K, phosphoinositide 3-kinase; QTc, corrected QT interval; RT, radiotherapy; SERM, selective estrogen receptor modulator; T-DM1, trastuzumab emtansine; T-DXd, trastuzumab deruxtecan; TNBC, triple-negative breast cancer; VTE, venous thromboembolism. The reported incidences, when available, are representative estimates and are dependent on the endpoint, drug, population, and follow-up duration; therefore, they should not be compared directly across treatment classes. The following references were used as supporting sources for the reported incidences [2,12,16,19,20,21,22,23,24].

## 2. microRNAs: Definition, Biogenesis, and Mechanisms of Action

microRNAs (miRNAs) are small non-coding RNAs (~22 nt) that act as key post-transcriptional regulators of gene expression [25,26,27]. Through a conserved 5′ seed region (nucleotides 2–7), miRNAs recognize target mRNAs and mediate gene silencing. This short sequence requirement enables individual miRNAs to regulate multiple targets, while single mRNAs can be modulated by numerous miRNAs, particularly through multiple binding sites within their 3′ untranslated regions. Moreover, related miRNAs are often organized in polycistronic clusters and co-transcribed, supporting coordinated regulation. Accordingly, miRNA biology is characterized by highly ordered, network-based control of both miRNA biogenesis and miRNA–target interactions [25,26,27].

**Figure 1:**
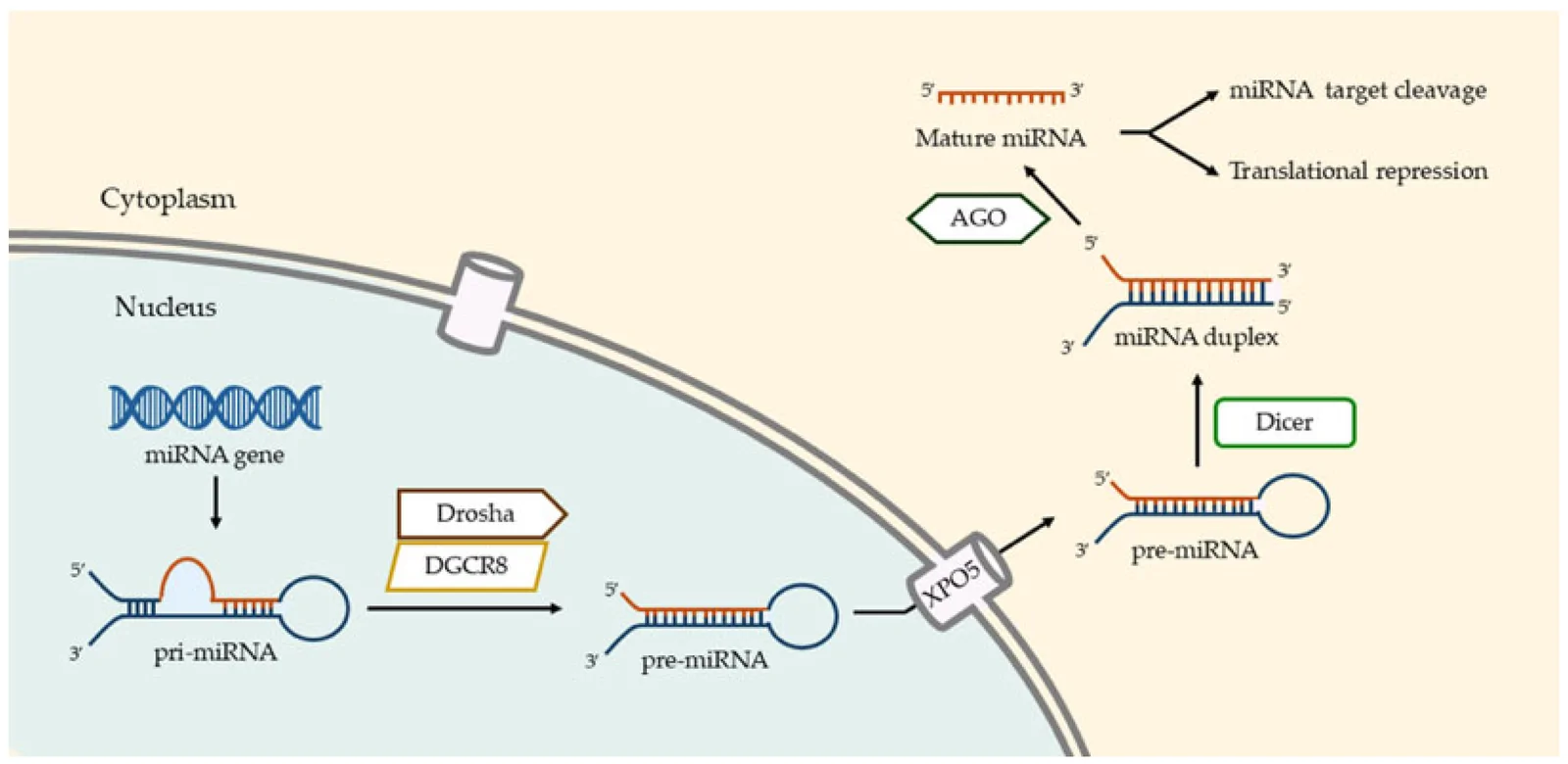
miRNA biogenesis. Primary miRNA transcripts (pri-miRNAs) are processed in the nucleus by the Drosha/DGCR8 microprocessor complex to generate precursor miRNAs (pre-miRNAs). Pre-miRNAs are exported to the cytoplasm through the Exportin-5 (XPO5) complex and subsequently cleaved by Dicer to produce a mature miRNA duplex comprising the 5p and 3p strands. The duplex is loaded into Argonaute (AGO1–4) proteins, and one strand is preferentially retained as the guide strand, whereas the complementary passenger strand is unwound and degraded. The mature miRNA–AGO complex then mediates either mRNA cleavage or translational repression through sequence-dependent recognition of target RNAs.

miRNA biogenesis (Figure 1) is controlled by either miRNA-specific promoters or by the transcription of host genes, as most miRNAs are located within introns. In addition, many miRNAs are organized in genomic clusters and co-transcribed as a single primary transcript, forming functional units analogous to polycistronic operons [28].

miRNA biogenesis is a tightly regulated, multistep process that controls gene expression at the post-transcriptional level and proceeds through both canonical and non-canonical pathways [29].

The canonical biogenesis pathway represents the primary route for miRNA processing. In this pathway, primary miRNA transcripts (pri-miRNAs) are transcribed from their genes and subsequently processed into precursor miRNAs (pre-miRNAs) by the Microprocessor complex, which comprises the RNA-binding protein DiGeorge Syndrome Critical Region 8 (DGCR8) and the RNase III enzyme Drosha [30]. DGCR8 recognizes specific motifs within the pri-miRNA, including N6-methyladenylated GGAC sequences [31], while Drosha cleaves the pri-miRNA at the base of its characteristic hairpin structure, generating a pre-miRNA with a two-nucleotide 3′ overhang [32].

Pre-miRNAs are then exported to the cytoplasm *via* the Exportin-5 (XPO5)/Ran-GTP complex and further processed by the RNase III endonuclease Dicer [30], which removes the terminal loop to produce a mature miRNA duplex [33]. The two strands of this duplex are designated according to their origin from the pre-miRNA hairpin: the 5p strand from the 5′ end and the 3p strand from the 3′ end. Both strands can be loaded into Argonaute (AGO) proteins (AGO1–4 in humans) in an ATP-dependent manner [34].

Generally, the strand with lower 5′ end stability or a 5′ uracil is preferentially retained as the guide strand, while the complementary passenger strand is unwound and degraded. Fully complementary passenger strands are cleaved by AGO2 and subsequently degraded, producing pronounced strand bias. In contrast, duplexes with central mismatches or associated with non-AGO2 proteins are passively unwound and degraded *via* alternative mechanisms [29].

Non-canonical miRNA biogenesis pathways utilize alternative combinations of canonical pathway proteins, including Drosha, Dicer, Exportin-5, and AGO2, and are generally classified as Drosha/DGCR8-independent or Dicer-independent. Drosha/DGCR8-independent pre-miRNAs, such as intron-derived mirtrons [35] or 7-methylguanosine (m7G)-capped pre-miRNAs, resemble Dicer substrates and can be exported directly to the cytoplasm, with m7G caps often causing a strong 3p strand bias [36]. Dicer-independent miRNAs, processed by Drosha from short hairpin RNA (shRNA) transcripts, require AGO2 for cytoplasmic maturation, which involves AGO2-mediated slicing of the 3p strand and 3′–5′ trimming of the 5p strand [37].

## 3. microRNA Involved in Cardiotoxicity, Modulated by Cancer Therapies of Skin and Breast Cancers

Cancer therapies in skin and breast cancers modulate different miRNAs. Their modulation has been linked to cardiotoxic effects, either by exacerbating them, counteracting them, or serving as biomarkers.

miRNAs have been classified based on their functions or expression in (i) OncomiRs—miRNAs that contribute to cancer development and progression by promoting tumorigenic processes; (ii) Tumor suppressor miRs—miRNAs that inhibit tumorigenesis by repressing oncogenic pathways; (iii) CardiomiRs—heart-specific function and cardiac disease; (iv) MyomiRs—muscle biology miRNA relevant in skeletal and cardiac muscle regulation; (v) AngiomiR, an endothelial-enriched miRNA that is relevant in vascular homeostasis; (vi) InflammomiR, miRNAs that participate in the regulation of inflammation and immune responses. Some of them belong to more classes depending on the context.

The following miRNAs, summarized in Table 2, are the most relevant.

### 3.1. Tumor Suppressor miRNAs

#### 3.1.1. miR-200 Family

The miR-200 family (miR-200) consists of five members (i.e. miR-200a, miR-200b, miR-200c, miR-141, and miR-429). This miRNA family has been extensively studied in the epithelial-to-mesenchymal transition (EMT) of tumor cells [66], but has now very well-established its role in oxidative stress and inflammatory mechanisms and hence in cardiac function [67]. The miR-200 family in skin tumors and melanoma is downregulated and promotes EMT phenotypic switching, invasion, metastasis, and resistance to therapy; indeed, restoration of miR-200 expression suppresses these malignant traits [68]. In breast cancer, miR-200 family is also downregulated; the predominant mechanism of miR-200 demise is epigenetic silencing through promoter DNA hypermethylation and PRC2/EZH2-mediated histone modifications that is reinforced by the ZEB1/ZEB2-miR-200 double-negative feedback loop, which drives EMT, metastasis, and therapy resistance [69].

Since tumor therapies modulate EMT, as a consequence, the miR-200 family plays a major role. In particular, DOX treatment has been shown to induce miR-200c in human cardiac mesenchymal progenitor cells (CmPCs) [9], and this increase is dependent on oxidative stress and DNA damage. Upregulation of miR-200c induces apoptosis and senescence in endothelial cells through targeting of the transcription factor ZEB1 [70]. In addition, it promotes oxidative stress and reduces nitric oxide bioavailability [71], disrupting the regulatory circuit involving endothelial nitric oxide synthase, sirtuin 1, and FOXO1 *via* directly targeting all of them [71]. In a mouse model of cardiotoxicity, it was shown that miR-200c was upregulated in the LV of mice and that the treatment with stromal cell-derived factor 1 (SDF1), a cardioprotector, decreases miR-200c and p53 upregulation and increases ZEB1 [38]. Consistent with these observations, p21 mRNA, transcriptionally activated by p53 and repressed by ZEB1, is significantly upregulated following DOX treatment. In contrast, its expression is attenuated upon SDF1 administration. Notably, SDF1 confers cardioprotective effects in DOX-treated mice, partially reversing pathological cardiac remodeling, evidenced by reductions in left ventricular end-diastolic volume, improvements in left ventricular ejection fraction (LVEF) and diastolic anterior wall thickness, restoration of left ventricular end-systolic pressure, and a decrease in dP/dt [38].

In contrast, miR-200a expression has been reported to be reduced in DOX-treated mice, as well as in the rat cardiomyoblast cell line H9c2 following DOX exposure [39]. It was shown that miR-200a attenuates oxidative stress and cardiomyocyte apoptosis without significantly impacting matrix metalloproteinases or inflammatory mediators in an acute DOX model. Mechanistically, this effect is mediated through targeting Kelch-like ECH-associated protein 1 (Keap1), leading to the activation of nuclear factor erythroid 2-related factor 2 (Nrf2) [39].

In another study, miR-200b was found to be induced in the hearts of DOX-treated mice [40]. Many anticancer therapies increase reactive oxygen species (ROS) production, which modulates the expression of miR-200 family members. While this ROS-dependent response contributes to suppressing tumor progression, it can simultaneously exert harmful effects on cardiac tissue, promoting cardiotoxicity.

Although the above-described studies seem to be in contrast, they highlight the important role of miR-200 family members in cardiovascular homeostasis affected by cancer treatments.

#### 3.1.2. Let-7 Family

Originally discovered in Caenorhabditis elegans, let-7 was one of the first miRNAs identified and the first shown to be evolutionarily conserved in humans [72,73]. The let-7 family includes multiple members originating from at least 12 genetic loci distributed across several chromosomes (9, 11, 19, 21, 22, and X). In humans, ten let-7 microRNAs have been identified: let-7a, b, c, d, e, f, g, i, miR-98, and miR-202. Although these miRNAs share a similar length and sequence homology, they have distinct regulatory effects on protein translation and physiological processes.

The let-7 family plays a central tumor-suppressive role in both breast cancer and melanoma, where its dysregulation is associated with tumor progression, stemness, invasion, metastasis, and therapy resistance through the modulation of oncogenic signaling pathways [74]. In melanoma, reduced let-7b expression contributes to malignant progression and impaired differentiation by targeting key cell-cycle regulators [75]. In breast cancer, let-7 members are also involved in endocrine and chemoresistance mechanisms: let-7b and let-7i regulate tamoxifen sensitivity through targeting ER-α36. At the same time, restoration of let-7 expression resensitizes resistant cells [76]. Moreover, let-7c-5p and let-7d have been implicated in taxane resistance and radio sensitization, respectively, *via* the modulation of key survival and proliferation pathways such as CCR7/SOCS1 and Cyclin D1/AKT1/Wnt1 signaling [76,77,78]. Additionally, let-7a regulates HER2 and PARP1 expression, linking its downregulation in HER2-positive breast cancer to potential therapeutic implications for anti-HER2 and PARP inhibitor strategies [79]. In melanoma, let-7a also modulates apoptotic responses to BRAF/MEK inhibitors, influencing treatment sensitivity [80]. Finally, let-7-mediated repression of PD-L1 further suggests a potential role in immune checkpoint regulation, with implications for immunotherapy response [81].

In the cardiac context, inhibition of let-7c has been shown to modulate post-myocardial infarction (MI) remodeling by increasing the expression of pluripotency-associated genes such as Sox2 and Oct4 in cardiac fibroblasts, both in vitro and in vivo. Let-7c antagonism reduced interstitial collagen deposition, limited apoptosis, and preserved systolic function after MI, suggesting its potential as a novel therapeutic strategy [82].

Different let-7 family members have been linked to CTRCD. Let-7f is involved in anthracycline-induced cardiotoxicity. Let-7f, together with other miRNAs, was significantly downregulated in patients treated with epirubicin who developed cardiotoxicity compared with patients without cardiotoxicity [83]. Moreover, let-7f levels are associated with subsequent cardiac impairment and correlate with established cardiac biomarkers, suggesting prognostic and predictive value for cardiotoxicity risk. Preclinical data showed that let-7g is downregulated in DOX-exposed rat hearts and correlates with functional and injury markers such as troponin T and hemodynamic parameters [42].

Overall, specific let-7 members may serve as potential biomarkers and modulators of chemotherapy-induced cardiac injury.

#### 3.1.3. miR-34 Family

The miR-34 family is composed of three closely related miRNAs: miR-34a, miR-34b, and miR-34c. miR-34a is encoded by a gene located on chromosome 1p36.22 within the MIR34A genomic locus; miR-34b and miR-34c are co-transcribed and located on chromosome 11q23.1. They are frequently deleted in several human cancers, including breast cancer and melanoma.

The miR-34 family is a direct target of the tumor suppressor p53. It exerts antitumor effects by promoting apoptosis, cell cycle arrest, and senescence through the downregulation of target genes such as CDK4, CDK6, and Bcl-2 [84,85]. Although miR-34 downregulation in tumors may result from alterations in p53 signaling, additional mechanisms may also contribute to its silencing. For example, in melanoma, miR-34 expression has been reported to be suppressed through CpG promoter methylation [86].

In breast cancer, low miR-34a expression has been associated with multidrug resistance (MDR) and poorer clinical outcomes. In MDR-MCF-7 cells, miR-34a is downregulated, while its restoration partially reverses drug resistance by reducing the expression of Bcl-2, CCND1, and NOTCH1 [87]. miR-34a is an important regulator of cardiovascular pathology and cardiac remodeling. It is upregulated in heart tissue in conditions such as MI and HF, where it contributes to cardiomyocyte apoptosis, oxidative stress, inflammation, and impaired autophagy. This increased expression is associated with cardiac cell death, reduced repair capacity, adverse ventricular remodeling, and progression toward heart failure with fibrosis and dysfunction. Overall, miR-34a is considered a key mediator of cardiac injury and a potential therapeutic target in cardiovascular diseases (CVDs) [88].

It has also been suggested that miR-34a is involved in resistance to taxane-based anticancer therapy. In particular, miR-34a promotes the downregulation of cyclin D1, thereby inducing G1 cell cycle arrest and reducing the cytotoxic efficacy of docetaxel, which mainly targets actively dividing cells, ultimately contributing to drug resistance in breast cancer cells [89].

miR-34a has been shown to help overcome resistance to CDK4/6 inhibitors in estrogen receptor-positive breast cancer. In this context, the inhibition of BET bromodomains can restore sensitivity to these drugs by increasing miR-34a levels. In turn, miR-34a reduces the expression of CDK6, leading to the reactivation of cell cycle arrest and improved response to therapy [90].

In NMSK, miR-34a is generally downregulated in tumor tissues compared to healthy skin. This reduction is associated with increased cell proliferation and decreased apoptosis, suggesting a role of miR-34a in tumor progression [91].

miR-34a is involved in DOX-induced cardiotoxicity by promoting oxidative stress and cardiomyocyte apoptosis. Particularly in rats, DOX treatment upregulates miR-34a expression, which suppresses SIRT1, which is a deacetylase that deacetylates the p66ShcA gene promoter [92]. p66Shc is a redox enzyme that promotes mitochondrial ROS generation and mediates oxidative stress signaling [93]. Thus, SIRT1 decrease activates the p66Shc pathway, ultimately contributing to cardiac injury. Importantly, inhibition of miR-34a showed cardioprotective effects, suggesting its potential as a therapeutic target to mitigate DOX-related cardiotoxicity [43].

miR-34b/c are upregulated by DOX-treatment in the murine adult cardiomyocyte cell line HL-1 [45]. miR-34b/c upregulation suppressed the E3 ubiquitin ligase ITCH, a negative regulator of NF-κB signaling resulting in enhanced inflammatory responses, increased TNF-α and IL-6 expression, and myocardial injury, while miR-34b/c inhibition mitigated cardiac damage and improved cardiomyocyte survival [45]. Moreover, miR-34a is upregulated in macrophages following anti-PD-1 inhibitor treatment used to treat non-melanoma skin cancer (NMSC), contributing to impaired cardiac function. This impairment occurs through promoting M1 macrophage polarization and inducing myocardial inflammation *via* the miR-34a/KLF4 signaling pathway. In particular, miR-34a promotes pro-inflammatory polarization by downregulating its target gene KLF4, thereby enhancing cardiac injury [44].

A Phase I study of MRX34, a liposomal miR-34a mimic administered twice weekly in patients with advanced solid tumors, was terminated early due to immune-related toxicities, and the overall development program was subsequently stopped (in September 2016) [94]. In fact, MRX34 can trigger immune toxicity since the immune system can mistake synthetic RNA molecules for viral genetic material, activating innate immune receptors, especially Toll-like receptors (TLRs) [94].

Further detailed analyses of immune-related adverse events and toxicity were conducted in advanced solid tumor patients.

Considering the detrimental role of miR-34a in cardiotoxicity, its *in vivo* administration warrants careful assessment.

#### 3.1.4. miR-29 Family

The miR-29 family includes three members: miR-29a, miR-29b, and miR-29c, which differ by only two or three nucleotides. miR-29b exists as two genomic variants, miR-29b1 and miR-29b2. The family is encoded by two gene clusters transcribed in tandem: MIR29B1/MIR29A located on chromosome 7 and MIR29B2/MIR29C located on chromosome 1 [95].

The miR-29 family is dysregulated in both breast cancer [96] and melanoma [97], where it is involved in tumor progression and cancer-related signaling pathways.

BRAF inhibitors are used to treat melanoma. It has been demonstrated that the oncogene BRAF paradoxically promotes the expression of miR-29b1/a in mouse embryonic fibroblasts (MEFs) and melanocytes, whereas MAPK signaling and p53 promote miR-29b2/c expression. The reduced expression of miR-29b2/c due to p53 inactivation leads to the upregulation of its targets, the oncogenes MAFG and MYBL2, thereby promoting melanoma progression. The authors also demonstrated the tumor-suppressive effects of miR-29 mimics *in vivo*, highlighting its potential therapeutic use [98,99].

Tamoxifen induces miR-29a and miR-29b-1 in endocrine-sensitive breast cancer cells, supporting its anti-proliferative effects. In contrast, this regulatory effect is reduced or absent in endocrine-resistant cells, linking the miR-29 control to treatment response. However, they do not seem to confer tamoxifen resistance in cancer cells; nevertheless, they have been observed to regulate lncRNAs associated with endocrine resistance in breast cancer [100,101].

miR-29b is also involved in the CDK4/6 inhibitor resistance that develops in breast cancer patients. In particular, it has been demonstrated that c-myc upregulation promotes miR-29b downregulation, CDK6 upregulation, and the inhibition of G1 cell-cycle arrest [102].

Members of the miR-29 family have emerged as important regulators of cardiac development and remodeling. In particular, miR-29b-3p is upregulated in the right ventricular outflow tract (RVOT) of patients with congenital heart disease, where it impairs cardiomyocyte proliferation by negatively regulating NOTCH2 [103].

The miR-29 family also plays a role in cardiac remodeling. miR-29, derived from cardiac myocytes, has been shown to promote pathological hypertrophy and fibrosis by modulating the Wnt pathway. Importantly, genetic deletion or the pharmacological inhibition of miR-29 prevents cardiac hypertrophy, reduces fibrosis, and improves cardiac function in models of pressure overload [104]. Furthermore, elevated circulating levels of miR-29a have been reported in patients with cardiac hypertrophy, and these levels are inversely correlated with the extent of cardiac fibrosis. Together, these findings show the important role of the miR-29 family in CVDs [105].

The miR-29 family is also involved in CTRCD. An increase in plasma miR-29b levels was observed in children and young adults treated with anthracycline chemotherapy (AC). Post-treatment plasma miR-29b expression was elevated, and a dose-dependent relationship was identified between AC exposure and markers of cardiac injury [46].

On the other hand, miR-29b was markedly reduced in the myocardium of DOX-treated rats [47]. Restoring the miR-29b levels in cardiac tissue significantly improved cardiac performance. In rat cardiomyocytes, miR-29b overexpression attenuated DOX-induced apoptosis, as miR-29b directly targets the anti-apoptotic protein Bax [47].

Radiotherapy (RT) is commonly used to treat breast and skin cancers. However, radiation can induce vasculopathy, increasing the risk of ischemic CVDs such as MI, HF, and stroke in a dose-dependent manner [24]. In arteries from patients undergoing microvascular free tissue transfer, a comparison between irradiated and non-irradiated vessels showed that irradiation reduced miR-29b expression. Both in vitro and *in vivo* experiments demonstrated that miR-29b overexpression therapy can reduce vascular inflammation by inhibiting pentraxin-3 and dipeptidyl peptidase-4, which are involved in the regulation of inflammatory responses and extracellular ligand interactions [48].

In another study, circulating miR-29c levels were downmodulated after RT exposure. They showed correlations with echocardiographic parameters indicative of early myocardial stress and subclinical cardiac remodeling, supporting its potential as a biomarker of radiation-induced cardiac injury [106].

Therefore, miR-29 downregulation in RT could predict a negative impact on CVDs.

#### 3.1.5. miR-30 Family

The miR-30 family includes miR-30a, miR-30b, miR-30c, miR-30d, and miR-30e, with miR-30c further subdivided into miR-30c-1 and miR-30c-2. These miRNAs are encoded by six genes located on human chromosomes 1, 6, and 8 [107]. Although all members share an identical seed sequence, they differ in their compensatory sequences at the 3′ end. This variation enables the miR-30 family to regulate distinct target genes and signaling pathways, thereby supporting a wide range of biological functions [107].

The miR-30 family is dysregulated in several cancers, including breast cancer and NMSC. In breast cancer, miR-30 increased expression has been associated with improved patient survival and better response to chemotherapy, suggesting its potential role as a prognostic and predictive biomarker [108].

miR-30b has been implicated in the modulation of trastuzumab response in HER2-positive breast cancer. Specifically, miR-30b directly targets and downregulates CCNE2 (cyclin E2), a cell-cycle regulator associated with trastuzumab resistance. Through CCNE2 suppression, increased miR-30b expression enhances the sensitivity of HER2-positive breast cancer cells to trastuzumab [109].

High levels of miR-30c have been associated with longer progression-free survival (PFS) in patients treated with tamoxifen for ER-positive breast cancer. A regulatory link between miR-30c and the ER-α/EGFR signaling axis may explain this clinical benefit. Tumors with higher miR-30c expression tend to show increased ER-α levels and reduced EGFR expression, whereas enhanced EGFR/HER2 signaling has been associated with resistance to endocrine therapy. Overall, elevated miR-30c may identify tumors that remain more dependent on estrogen signaling and therefore more are responsive to tamoxifen-based endocrine treatment [110].

miR-30c is also involved in chemotherapy resistance. Indeed, it has been demonstrated that the over-expression of miR-30c leads to an increase in paclitaxel resistance in a triple-negative breast cancer cell line, whereas the inhibition of miR-30c can restore drug sensitivity [111].

It has been demonstrated that in NMSC patients, circulating miR-30e is significantly upregulated, whereas circulating miR-30a is downregulated, compared with healthy controls. These findings suggest that members of the miR-30 family may serve as non-invasive biomarkers for NMSC detection [112].

The miR-30 family is highly expressed in cardiac tissue but is generally downregulated under pathological conditions such as cardiomyocyte hypertrophy and MI [113].

miR-30a showed significantly higher circulating levels in patients who later developed left ventricular dysfunction and HF. Importantly, elevated miR-30a at admission was associated with adverse cardiac remodeling and reduced left ventricular ejection fraction at follow-up [114].

AC treatment with DOX, used in breast cancer, induces the downregulation of miR-30 family members in rat cardiomyocytes. This effect is mediated by GATA-6 upregulation, leading to the deregulation of key cardiac genes, contributing to DOX-induced cardiotoxicity and enhancing apoptosis [49].

Given the critical involvement of the miR-30 family in cancer progression and cardiovascular homeostasis, alterations in miR-30 expression may influence both the therapeutic response to anticancer treatments and the development of cardiovascular toxicity.

#### 3.1.6. miR-140-5p

Among the two mature products generated from the pre-miR-140 precursor, miR-140-5p is generally considered the predominant and most extensively studied strand. However, miR-140-3p also exhibits biological activity and contributes to the regulation of distinct cellular processes. miR-140-5p plays an important role in regulating cell proliferation, apoptosis, senescence, and inflammatory responses. Aberrant expression of miR-140-5p has been implicated in the pathogenesis of various cancers and age-related disorders, and its circulating levels are being explored as potential biomarkers for predicting disease progression [115].

In melanoma, miR-140-5p is frequently downregulated and exerts tumor-suppressive effects. By directly targeting SOX4, miR-140-5p inhibits cell proliferation, invasion, and tumor growth, while attenuating oncogenic Wnt/β-catenin and NF-κB signaling pathways [116].

In breast cancer, miR-140-5p acts as a tumor suppressor by inhibiting proliferation, migration, invasion, cell-cycle progression, and tumor growth through suppression of the AKT/STAT3/NF-κB signaling pathway, accompanied by reduced CDK2 expression and increased E-cadherin levels [117]. Circulating miR-140-5p has been linked with predicting response to trastuzumab-based HER2-targeted therapy in breast cancer patients. When combined with miR-148a-3p, miR-140-5p improves the ability to identify patients who achieve a pathological complete response. Its predictive value is linked to biological pathways involved in cell metabolism and signaling networks such as AMPK and MAPK. Overall, miR-140-5p may serve as part of a circulating biomarker panel for therapy response prediction [118].

The function of miR-140-5p has been demonstrated in various CVDs. In a mouse model of atherosclerosis, miR-140-5p was upregulated, thereby increasing oxidative stress by suppressing the Nrf2/Sirt2/HO-1 antioxidant pathway. On the contrary, its inhibition reduced ROS production and oxidative damage, suggesting a pathogenic role in atherosclerosis-associated hypertension [119].

Moreover, miR-140-5p is upregulated in atherosclerotic plaque tissues and ox-LDL-treated vascular smooth muscle cells, where it promotes cell proliferation, migration, and invasion while inhibiting apoptosis through the direct suppression of roundabout guidance receptor 4 (ROBO4), an endothelial-specific receptor that maintains vascular integrity by stabilizing blood vessels and regulating angiogenesis and endothelial barrier function. Therefore, the upregulation of miR-140-5p, which causes ROBO4 downregulation, contributes to disease progression [119].

In an in vitro atrial fibrillation (AF) model, HL-1 cardiomyocytes subjected to rapid electrical pacing (5 Hz for 24 h) exhibited a marked downregulation of miR-140-5p expression relative to the control cells, suggesting a potential protective role for this miRNA in AF pathogenesis. miR-140-5p alleviates AF-associated cardiomyocyte injury by directly targeting and downregulating aquaporin 4 (AQP4), thereby reducing inflammation, apoptosis, and adverse electrical remodeling in cardiac cells [120].

In a rat model of myocardial ischemia-reperfusion injury, miR-140-5p is upregulated, and anti-miR-140-5p treatment improves cardiac function, reduces myocardial infarct area, and myocardial cell apoptosis. Moreover, in the hypoxia/reoxygenation (H/R) model in the rat embryonic ventricular cardiomyoblast cell line H9c2, miR-140-5p is upregulated. It promotes cardiomyocyte apoptosis, worsening cardiomyocyte function by directly targeting and downregulating the anti-apoptotic protein BCL2L1 [121].

miR-140-5p plays a major role in CTRCD; it is upregulated in a rat model of DOX-induced cardiotoxicity and also contributes to cardiac injury. It promotes myocardial oxidative stress by directly targeting and suppressing Nrf2 and Sirt2, key regulators of antioxidant defense. As a consequence, there is an increase in ROS production and worsening of DOX-induced heart damage. Modulating miR-140-5p levels may therefore represent a potential therapeutic strategy against cardiotoxicity [50].

#### 3.1.7. miR-144

miR-144 is a tumor suppressor miRNA but is also classified as an “inflammamiR” due to its involvement in regulating immune and inflammatory pathways. By modulating the expression of inflammatory mediators and signaling molecules, it fine-tunes both pro-inflammatory and anti-inflammatory responses. However, its expression and biological effects may vary according to the pathological context [122].

miR-144 is commonly downregulated in melanoma, where it acts as a tumor suppressor by targeting oncogenic pathways, including SMAD1 and c-Met signaling. Through these mechanisms, miR-144 inhibits melanoma cell proliferation, migration, invasion, and metastasis, highlighting its potential as a therapeutic target and biomarker of disease progression [123].

miR-144 is also downregulated in breast cancer, and low levels of miR-144 are associated with poor differentiation, higher clinical stage, metastasis, and EMT through targeting of ZEB1/ZEB2, transcription factors that regulate oncogenic signaling pathways. Restoration of miR-144 suppresses breast cancer progression and increases sensitivity to anticancer therapies [124].

Beyond its role in tumor suppression and inflammation, miR-144 regulates lipid metabolism primarily by directly targeting ABCA1, a key transporter involved in cholesterol efflux and HDL biogenesis, thereby influencing reverse cholesterol transport and systemic lipid homeostasis. In addition, miR-144 modulates oxidative stress responses through targeting Nrf2, a master regulator of antioxidant defense pathways [125]. Through its combined effects on metabolic and inflammatory signaling, miR-144 is considered a component of the regulatory network connecting lipid dysregulation and redox imbalance with both cancer development and atherosclerotic cardiovascular disease (ASCVD) [126].

In a rat model of DOX-induced heart failure and in H9C2 cardiomyocytes exposed to DOX, miR-144-3p was downregulated, contributing to cardiac injury through reduced cell viability, increased apoptosis, and impaired PI3K/AKT signaling. Overexpressing miR-144-3p alleviated cardiac dysfunction and cardiomyocyte apoptosis by directly targeting suppressor of cytokine signaling 2 (SOCS2), therefore reactivating the PI3K/AKT pathway, highlighting a protective role for miR-144-3p in anthracycline-induced heart failure [51].

In the context of RT, miR-144 has been proposed as a modulator of treatment response by influencing tumor cell sensitivity to ionizing radiation (IR). Its functional role appears to be highly context-dependent, as it may act either as a tumor suppressor or as a contributor to therapy resistance, depending on the cellular and molecular milieu. Accordingly, miR-144 has emerged as a putative biomarker and therapeutic regulator in cancer management [127].

Given its involvement in oxidative stress regulation and inflammatory signaling, miR-144 dysregulation, which contributes to impaired redox homeostasis and enhanced inflammatory and apoptotic signaling, may be relevant to CTRCD and potentially exacerbate it.

#### 3.1.8. miR-146a

miR-146a is a member of the miR-146 family and may exert either tumor-suppressive or oncogenic functions depending on the cancer type and cellular context [128]. It inhibits proliferation, migration, and invasion in several cancers, including gastric, lung, breast, and pancreatic tumors [128,129,130,131], while promoting tumor progression in anaplastic thyroid cancer and melanoma [132]. Moreover, miR-146a is another “inflammomiR” that negatively regulates inflammation-induced immune responses through the NF-κB pathway and has been associated with oncogenic transformation [133]. Therefore, modulation of miR-146a expression or function may provide important insights into cancer progression and represent a potential therapeutic strategy.

In addition, miR-146a has been identified as a protective factor in several CVDs, mainly due to its anti-inflammatory and anti-atherogenic properties. Specifically, miR-146a negatively regulates the IRAK1/TRAF6/NF-κB signaling pathway, thereby reducing vascular inflammation, macrophage activation, foam cell formation, and atherosclerotic plaque instability [134,135,136,137]. Furthermore, several studies have demonstrated that miR-146a contributes to the endothelial protection and attenuation of myocardial injury, although its effects may vary with the pathological context and cell type.

Furthermore, it has been shown that miR-146a exerts cardioprotective effects during DOX treatment in human cardiomyocytes by reducing apoptosis, modulating autophagy, and suppressing inflammatory signaling pathways. In particular, miR-146a targets the TAF9b/p53 signaling axis, thereby reducing p53-mediated apoptosis and the expression of inflammatory mediators such as IRAK1 and TRAF6 [52,53]. Consistently, overexpression of miR-146a decreases cardiac injury in experimental models of chronic DOX exposure [52].

However, the role of miR-146a is not exclusively protective. Some studies have demonstrated that DOX-induced upregulation of miR-146a may suppress the neuregulin-1/ErbB signaling pathway by directly targeting ErbB4, a pathway essential for cardiac survival and repair. In particular, studies in murine hearts have shown that excessive miR-146a expression may contribute to acute cardiotoxicity, especially during combined treatment with trastuzumab [53].

### 3.2. OncomiR

#### 3.2.1. miR-21

miR-21 is located on chromosome 17q23.2, within a genomic region that overlaps with the protein-coding gene TMEM49. It is widely expressed across multiple tissues, including the heart.

miR-21 is consistently upregulated in many cancers, where it acts as an oncomiR.

In breast cancer, circulating plasma levels of miR-21 are significantly higher in patients compared with healthy individuals and patients with benign breast conditions. In addition, miR-21 is upregulated in cancer cells, with its expression correlating with tumor stage and aggressiveness. Functionally, miR-21 promotes breast cancer cell proliferation and metastasis by targeting LZTFL1. These findings suggest that plasma miR-21 may serve as a promising biomarker for both diagnosis and prognosis in breast cancer [138].

miR-21 promotes melanoma progression by enhancing tumor cell invasiveness through the downregulation of TIMP3, a key inhibitor of metalloproteinases that degrade the extracellular matrix. As a result, increased miR-21 expression facilitates tumor invasion and metastasis. Importantly, the inhibition of miR-21 in vivo reduces melanoma invasiveness, suggesting its potential as a therapeutic target in melanoma [139].

miR-21 is involved in resistance to BRAF inhibitors used in melanoma treatment. When miR-21 is present at high levels, melanoma cells become more aggressive and less responsive to therapy since miR-21 reduces the activity of tumor-suppressor genes and activates alternative survival pathways, especially the PI3K/AKT pathway [140]. Its involvement in these mechanisms also suggests a role in resistance to MEK inhibitors, since miR-21-driven activation of compensatory survival signaling contributes to the reduced effectiveness of MAPK pathway-targeted therapies [141].

miR-21 has been associated with resistance to AC-based chemotherapy. In particular, it is frequently overexpressed in resistant cancer cells, where it contributes to reduced drug sensitivity by inhibiting pathways involved in apoptosis, cell cycle regulation, and cell survival, thereby promoting tumor cell proliferation and survival. As a result, miR-21 is considered a potential biomarker of AC resistance and a possible therapeutic target to improve treatment response [142].

miR-21 is also upregulated in trastuzumab-resistant HER2-positive breast cancer, where it contributes to reduced therapeutic efficacy. This effect is mediated by the suppression of PTEN, which leads to activation of the PI3K/AKT signaling pathway, which promotes cell survival and proliferation [143].

miR-21 is also involved in resistance to therapy with tamoxifen in breast cancer. In particular, high levels of miR-21 are associated with reduced drug sensitivity, while its silencing restores responsiveness to treatment. This effect is mediated by the inhibition of the PI3K/AKT/mTOR signaling pathway, which leads to increased cell death and enhanced antitumor efficacy of tamoxifen [144].

miR-21 expression in tumor-associated macrophages plays a role in regulating the response to anti-PD-1 immunotherapy in melanoma. High levels of miR-21 promote an immunosuppressive macrophage phenotype that reduces antitumor immune activity. Conversely, depletion of miR-21 enhanced the ability of macrophages to kill cancer cells and improved the efficacy of PD-1 blockade [145].

miR-21 is expressed at higher levels in cardiac fibroblasts than in cardiomyocytes and is significantly upregulated in fibroblasts from failing hearts. In response to cardiac stress, increased miR-21 levels enhance ERK–MAPK signaling, promoting fibroblast proliferation and contributing to cardiac fibrosis. Conversely, inhibition of miR-21 reduces ERK–MAP kinase activity by upregulating its target sprouty homolog 1 (Spry), thereby decreasing interstitial fibrosis and improving cardiac function [146].

In experimental models of acute myocardial infarction (AMI), miR-21 is downregulated in infarcted myocardial regions while upregulated in border zones at 6 and 24 h after injury. Importantly, miR-21 overexpression has been shown to reduce infarct size 24 h after AMI [147]. In experimental models of AMI, miR-21 exerts cardioprotective effects by directly repressing programmed cell death 4 (PDCD4), thereby relieving its inhibitory control on activator protein 1 (AP-1)-dependent signaling and promoting a downstream transcriptional program that promotes cell survival and attenuates cardiomyocyte apoptosis, ultimately contributing to reduced infarct size [148].

In chronically DOX-treated mice, miR-21 is upregulated in the myocardium, whereas it is not significantly modulated following acute DOX treatment [54]. Upon DOX treatment, miR-21 was upregulated in both cardiac tissue and H9C2 cardiomyocytes and attenuated myocardial injury by suppressing apoptosis through direct targeting of the stress-responsive pro-apoptotic protein B-cell translocation gene 2 (BTG2) [54].

miR-21 also plays a key role in radiation-induced toxicity, as it is upregulated by IR in human fibroblasts and in the myocardium of irradiated rats [55]. Its induction has been associated with the modulation of extracellular matrix remodeling and protein kinase C (PKC) signaling, potentially affecting cardiac electrical coupling through connexin 43 (Cx43) [58].

Since miR-21 upregulation appears to exert damaging effects in cardiac tissue, its inhibition may help attenuate the cardiotoxicity associated with various anticancer therapies.

#### 3.2.2. miR-210

miR-210 is one of the best-characterized hypoxia-associated miRNAs and is strongly induced under low oxygen conditions through the activation of HIF-1α. It regulates multiple biological processes, including mitochondrial metabolism, angiogenesis, apoptosis, cell proliferation, DNA repair, oxidative stress response, and cellular adaptation to hypoxia [149,150,151].

Under hypoxic conditions, HIF-1α directly promotes miR-210 expression, which in turn modulates cellular survival pathways and metabolic adaptation. In particular, miR-210 suppresses mitochondrial respiration by targeting genes such as ISCU, SDHD, and COX10, thereby promoting a metabolic shift toward glycolysis and reducing oxidative stress [149,152,153].

In cancer, miR-210 is frequently overexpressed and is generally associated with tumor progression, metastasis, therapy resistance, and poor prognosis. However, depending on the tumor type and cellular context, miR-210 may also exert tumor-suppressive effects [149,154,155].

miR-210 is consistently upregulated in hypoxic tumors and functions as an oncomiR, regulating mitochondrial metabolism, angiogenesis, and survival pathways in cancers including breast cancer and melanoma. It promotes metabolic reprogramming, invasion, and metastatic progression by regulating mitochondrial and hypoxia-related target genes. Its increased expression in plasma correlates with poor prognosis and disease [156,157].

miR-210 also plays a crucial role in angiogenesis by enhancing endothelial cell migration, proliferation, survival, and neovascularization, thereby supporting tissue repair in ischemic myocardium. Furthermore, it contributes to the inhibition of hypoxia-induced apoptosis through the regulation of apoptosis-related targets, including AIFM3 and CASP8AP2, ultimately promoting cardiomyocyte survival and limiting myocardial damage following ischemic injury [149,158]. Experimental evidence suggests that miR-210 attenuates adverse cardiac remodeling and improves cardiac function after myocardial infarction, supporting its potential therapeutic role in CVDs. Accordingly, miR-210 is widely recognized as a cardioprotective miRNA, particularly under hypoxic and ischemic conditions [150,152,158].

Several studies evaluating pro-angiogenic miRNAs in AC-induced cardiotoxicity among breast cancer patients have shown that miR-210 is significantly downregulated in patients who develop epirubicin-related cardiotoxicity compared with those in the non-cardiotoxicity group [41,83]. Moreover, miR-210 levels negatively correlate with cardiac injury biomarkers, such as cTnI and NT-proBNP [83].

These findings suggest that miR-210 may be associated with AC-induced cardiotoxicity in breast cancer patients.

### 3.3. MyomiRs/CardiomiRs

#### 3.3.1. miR-1

miR-1 is encoded by the two genes MIRN1-1 and MIRN1-2, located on chromosome 18 [159].

miR-1 functions as a tumor suppressor in breast cancer, with reduced expression observed in tumor tissues and cell lines. Restoration of miR-1 inhibited breast cancer cell proliferation, migration, and invasion by directly targeting K-Ras (repressing oncogenic signaling) and interacting with the long non-coding RNA MALAT1, which sequester miR-1 as a competitive RNA to promote tumor growth [160].

It is highly expressed in the skeletal muscle and heart, and is deeply involved in its development and CVDs [161].

Indeed, miR-1 is upregulated in experimental models of MI and in patients with coronary artery disease (CAD). Mechanistically, miR-1 promotes arrhythmogenesis by repressing the translation of key cardiac ion channel genes, including GJA1 and KCNJ2, without substantially altering their mRNA levels. Inhibition of endogenous miR-1 has been shown to attenuate ischemia-induced arrhythmias, highlighting its potential as a therapeutic target in antiarrhythmic strategies [162]. Furthermore, circulating miR-1 levels are upregulated in acute MI. In patients, an association between miR-1 and creatine kinase-MB (CK-MB) levels has been reported [163]. Recent studies have demonstrated an increase in circulating miR-1 in patients with acute HF compared to healthy controls [164].

miR-1 has also been linked to AC-induced cardiotoxicity. Increased plasma levels of miR-1 were observed in DOX-treated breast cancer patients who developed cardiotoxicity compared with those without cardiac complications. miR-1 levels were associated with changes in LVEF and were useful to discriminate patients with cardiotoxicity better than cTnI levels, underlying its role as a biomarker for DOX-induced cardiotoxicity [56,57].

miR-1 is a critical regulator of cardiac electrical coupling and cell-to-cell communication through its targeting of Cx43 [165]. In rats subjected to a single dose of IR, downregulation of miR-1 was observed accompanied by increased Cx43 expression, which in turn enhanced myocardial intercellular communication, suggesting a compensatory cardioprotective response [58].

Taken together, current evidence suggests that circulating miR-1 may serve as a biomarker of anthracycline-induced cardiotoxicity. In contrast, decreased myocardial miR-1 expression after irradiation may represent a favorable adaptive cardiac response to radiation exposure.

#### 3.3.2. miR-133a

miR-133a is a muscle-specific microRNA highly expressed in cardiac and skeletal muscle [166].

In melanocytes to primary melanoma and from primary melanoma to metastatic melanoma cell lines, miR-133a has been found to be upregulated [167]. Moreover, miR-133a was significantly downregulated in breast cancer tissues and cell lines, and its reduced expression was associated with advanced disease stage, lymph node metastasis, and poorer patient survival. Conversely, miR-133a restoration inhibited breast cancer cell proliferation, migration, and invasion in vitro in breast cancer cells, supporting a potential tumor-suppressive role [168].

miR-133a is involved in the regulation of cardiomyocyte survival, hypertrophy, fibrosis, and electrical remodeling. miR-133a contributes to myocardial homeostasis and has attracted considerable interest as a potential biomarker of cancer therapy-related cardiotoxicity [166].

miR-133a is primarily involved in regulating cardiac hypertrophy. Reduced expression of miR-133a promotes hypertrophic remodeling through the upregulation of several targets, including calcineurin, NFATc4, Rac1, and Cdc42, which activate pro-hypertrophic MAPK signaling pathways [147]. In parallel, miR-133a exerts anti-apoptotic effects by suppressing caspase-9, thereby limiting cardiomyocyte death during stress conditions [169].

Following myocardial infarction, circulating exosomal miR-133a increases even before troponin T elevation [170]. Circulating miR-133a concentrations increased over time, displaying a trend similar to that of troponin I in the corresponding samples [171].

In experimental models of anthracycline cardiotoxicity, circulating miR-133a levels were found to increase after exposure to DOX, suggesting its release during myocardial injury. However, early studies did not observe a direct correlation with the exact onset of cardiac dysfunction [172].

This study identified circulating miR-133a as a potential early biomarker of DOX-induced cardiotoxicity in breast cancer patients. miR-133a was significantly upregulated in patients with cardiac injury. It was linked to dysregulation of the cardioprotective ErbB2 (HER2)/neuregulin-1 signaling pathway, suggesting a role in the mechanisms underlying chemotherapy-related cardiac damage [59].

In an interesting study, partial-body irradiation was shown to induce significant cardiac remodeling in the non-irradiated (out-of-field) heart, characterized by the activation of profibrotic signaling and development of myocardial fibrosis. Among the dysregulated microRNAs, miR-133a is notably downregulated. This loss is associated with enhanced fibrotic gene expression, including the upregulation of extracellular matrix genes such as collagen (COL)1A1 and COL3A1, fibronectin, and profibrotic mediators like connective tissue growth factor (CTGF) and transforming growth factor (TGF)-β1-related signaling components, thereby contributing to myocardial fibrosis. This study suggests that miR-133a plays a key protective role in limiting radiation-induced cardiac fibrosis even when the heart is not directly exposed [173].

Overall, miR-133a appears to act as a cardioprotective regulator by limiting hypertrophy, apoptosis, and fibrosis, while its circulating increase may reflect cardiomyocyte injury induced by oncologic therapies. Its monitoring may therefore offer a useful strategy for the early detection of treatment-related cardiotoxicity in patients receiving anticancer therapy.

#### 3.3.3. miR-208a/b

miR-208a and miR-208b are intronic microRNAs encoded within the myosin heavy chain (MHC) genes: miR-208a is located within the α-myosin heavy chain gene (Myh6), whereas miR-208b is embedded within the β-myosin heavy chain gene (Myh7). In the adult mouse heart, α-MHC/Myh6 and its intronic miR-208a are the predominant isoforms, whereas in the healthy adult human heart, β-MHC/Myh7 and miR-208b are predominantly expressed.

The miR-208 family plays a pivotal role in regulating the MHC isoform switch during cardiac development and under pathological conditions.

In melanoma, lncRNA MEG3 acts as a tumor suppressor by inhibiting malignant cell proliferation, invasion, and metastatic potential. Mechanistically, MEG3 regulates the miR-208/SOX4 signaling axis, limiting the oncogenic activity of miR-208 and reducing SOX4-mediated transcriptional programs involved in tumor progression and cancer stem cell maintenance. Restoration of miR-208 expression or SOX4 inhibition modulates MEG3-mediated effects, confirming the central role of this pathway in melanoma suppression [174].

Current evidence suggests that miR-208a acts as an oncogenic miRNA in breast cancer. Functional studies have shown that miR-208a promotes breast cancer stem cell self-renewal and tumor progression by establishing a positive feedback loop involving SOX2, β-catenin, LIN28, DICER1, and the let-7 family, thereby sustaining stemness and malignant phenotypes. Inhibition of miR-208a suppresses these effects, supporting its role in breast cancer progression [175].

In DOX-treated mice, cardiac miR-208a expression is upregulated and promotes cardiomyocyte apoptosis. Therapeutic silencing of miR-208a increases the expression of its target protein GATA4, a transcription factor that positively regulates the anti-apoptotic gene Bcl-2. Consequently, miR-208a inhibition attenuates DOX-induced cardiomyocyte apoptosis and is associated with improved cardiac function, as demonstrated by cardiac imaging analyses [60].

Regarding its biomarker potential, circulating miR-208a levels were reported to be elevated in a rat model of DOX-induced cardiotoxicity, suggesting its possible utility as a plasma biomarker of cardiac injury [176]. However, circulating miR-208a was undetectable in breast cancer patients who developed DOX-induced cardiotoxicity, indicating limited clinical applicability in this setting [150]. Interestingly, not all studies reported consistent changes in cardiac miR-208a expression. In one rat study, miR-208a levels progressively declined during cumulative DOX treatment, paralleling the downregulation of its host gene Myh6, whereas miR-208b expression was significantly increased [61]. Similarly, miR-208b was found to be upregulated in the hearts of DOX-treated mice [40], and several studies have also reported alterations in circulating miR-208b levels, further supporting its potential involvement in the cardiac response to anthracycline-induced injury.

Collectively, these studies identify miR-208a/b as a multifunctional stress-responsive miRNA involved in both anthracycline-induced cardiotoxicity and radiotherapy response, highlighting its promise as a therapeutic target while underscoring the need for further validation of its clinical utility as a circulating biomarker.

#### 3.3.4. miR-499

The miRNA-499 family is encoded within the myosin gene family and is located in an intron of the Myh7b gene. It includes three highly homologous members: miR-499a-5p, miR-499a-3p, and miR-499b.

The role of miR-499 in cancer appears to be context-dependent. In breast cancer, miR-499a-5p has been reported to be downregulated, resulting in upregulation of its target gene TMEM189, a key enzyme involved in plasmalogen biosynthesis, a class of ether phospholipids that maintain membrane integrity, regulate cellular signaling, and influence oxidative stress and ferroptosis susceptibility. Increased TMEM189 expression suppresses ferroptosis by modulating membrane lipid composition, thereby promoting breast cancer cell survival and progression [177].

In melanoma, miR-499-5p is upregulated and has been reported to promote tumor progression by targeting the deubiquitinase cylindromatosis (CYLD), a tumor suppressor involved in NF-κB signaling, thereby reducing its expression and enhancing melanoma cell proliferation and invasion. Conversely, long non-coding RNA MEG3 has been associated with poor prognosis. MEG3 functions as a competing endogenous RNA (ceRNA), acting as a molecular sponge for miR-499-5p. By sequestering miR-499-5p, MEG3 relieves its inhibitory effect on CYLD, leading to increased CYLD expression and the suppression of melanoma cell growth and invasiveness [178].

This family plays an important role in inhibiting cardiomyocyte progenitor cell proliferation and promoting their differentiation [179].

miR-499 is found at elevated levels in the plasma of patients with AMI, suggesting its potential as a novel diagnostic biomarker. It has been demonstrated that circulating miR-499 is significantly higher in AMI patients compared with non-AMI individuals and healthy controls. It can be detected as early as 1 h after the onset of chest pain, with levels progressively increasing within the first 9 h [179,180].

In anthracycline-treated breast cancer patients, circulating miR-499 is elevated in association with acute myocardial injury and correlates with cardiac troponin T levels, suggesting its potential utility as a biomarker of chemotherapy-related cardiotoxicity [62].

On the contrary, downregulation of miR-499-5p in the cardiomyocytes of mice exposed to DOX was observed, whereas an increase was found in serum. miR-499-5p decrease is responsible for mitochondrial fission and apoptosis, at least in part through up-regulation of its target p21 [63].

### 3.4. AngiomiR

#### 3.4.1. miR-126

miR-126 is encoded within the seventh intron of the epidermal growth factor-like domain 7 (EGFL7) gene on chromosome 9 and is co-transcribed with its host gene, leading to coordinated expression.

miR-126 is significantly downregulated in breast cancer, where it acts as a tumor suppressor by inhibiting cell migration and invasion through regulation of the PI3K/AKT signaling pathway and targeting genes such as ADAM9 [181]. Reduced miR-126 expression has also been associated with trastuzumab resistance in breast cancer cells. In trastuzumab-resistant models, miR-126 downregulation increased the expression of its target gene, Phosphoinositide-3-Kinase Regulatory Subunit 2 (PIK3R2), a crucial regulatory subunit in the PI3K/AKT signaling pathway, thereby activating the pathway. Conversely, restoration of miR-126 expression or silencing of PIK3R2 increased sensitivity to trastuzumab, highlighting the role of this axis in therapeutic resistance [182].

High miR-126 expression has been associated with improved therapeutic outcomes in ER-positive breast cancer, including a lower risk of recurrence and longer relapse-free survival following tamoxifen treatment [183]. In addition, miR-126 has emerged as a potential predictor of response to CDK4/6 inhibitors, likely through its regulation of cell-cycle progression and glycolytic pathways [184], highlighting its potential utility as a biomarker for endocrine and targeted therapies in breast cancer.

In melanoma, reduced miR-126 expression has been associated with resistance to BRAF inhibitors such as dabrafenib. Loss of miR-126 leads to the increased expression of oncogenic targets, including ADAM9 and VEGF-A, promoting tumor proliferation and invasion, whereas the restoration of miR-126 suppresses these processes by inducing G0/G1 cell-cycle arrest and inhibiting ERK1/2 signaling [185].

Combining miR-126 with an inhibitor of the PI3K/AKT signaling pathways in nanoparticles in metastatic melanoma resistant to BRAF, tumor cell proliferation and metastasis were reduced. Nanoparticles carrying a stabilized form of miR-126 showed promising therapeutic efficacy in vivo, suggesting a potential strategy to overcome drug resistance in advanced melanoma [186].

miR-126 is abundantly present in highly vascularized tissues such as the heart, lungs, and liver, where it contributes to the maintenance of endothelial homeostasis and vascular integrity [187].

Circulating miR-126 levels are significantly reduced in patients with CAD, with an even more pronounced decrease in individuals presenting with acute MI. miR-126 can also serve as a biomarker for assessing vascular obstruction and reperfusion in ischemic occlusive diseases. Notably, circulating levels of miR-126 within the first 24 h of symptom onset have emerged as a highly sensitive diagnostic and prognostic indicator for stroke patients [187].

Across two independent epirubicin/cyclophosphamide followed by docetaxel (EC-D) chemotherapy cohorts, circulating miR-126 was consistently reduced in breast cancer patients who developed cardiotoxicity and negatively correlated with cardiac injury biomarkers, suggesting a protective role of miR-126 in maintaining endothelial and vascular integrity during AC/taxane chemotherapy [41].

In a mouse study, it has been shown that radiation exposure increases endothelial extracellular vesicles (EVs) enriched in miR-126-5p, which in turn activate monocytes promoting vascular inflammation, suggesting a key role for EV-associated miRNAs in early radiation-induced vascular injury and their potential as biomarkers of radiation-related atherosclerosis [64].

Overall, these findings suggest that miR-126 plays a pivotal role in maintaining endothelial and vascular homeostasis; thus, its dysregulation contributes to both chemotherapy- and radiation-induced vascular injury, underlying its potential role as a biomarker and mediator of cardiovascular toxicity in cancer therapies.

#### 3.4.2. miR-130a

miR-130a is encoded on chromosome 11 and derives from a conserved miRNA locus involved in vascular and cardiac regulation [188].

miR-130a is upregulated in breast cancer, where it exerts a pro-oncogenic role by targeting the tumor suppressor PTEN. Increased miR-130a expression reduces the PTEN levels, enhances AKT phosphorylation, and promotes breast cancer cell growth, supporting tumor progression [189]. Its involvement in this signaling pathway suggests that miR-130a may also contribute to resistance to PI3K inhibitor-based therapies in breast cancer. miR-130b is also involved in the same regulatory axis and has been associated with breast cancer drug resistance. In particular, miR-130b targets PTEN and contributes to resistance to PI3K inhibitor-based therapies in breast cancer through activation of the PI3K/Akt signaling [190].

miR-130a is expressed in cardiovascular tissues and has been implicated in both cardiac remodeling and vascular pathology.

Circulating miR-130a shows stage-dependent changes in CVD. In pre-atherosclerotic patients, its levels are markedly reduced. In contrast, in established atherosclerosis and the peripheral blood of affected patients, miR-130a is significantly increased, indicating a dynamic regulation during disease progression [191].

In the heart, miR-130a appears to participate in adaptive and maladaptive responses to stress, with evidence showing that its expression is significantly upregulated in failing human hearts compared with non-failing controls, suggesting a role in the progression of HF [192].

Modulation of miR-130a has been implicated in AC-induced cardiotoxicity. DOX treatment induces the upregulation of miR-130a in mouse cardiomyocytes, thereby contributing to cardiac injury by suppressing PPARγ signaling. The latter is involved in attenuating oxidative stress, inflammation, apoptosis, and fibrosis, thereby preserving mitochondrial function. Conversely, inhibition of miR-130a attenuates DOX-induced cardiotoxicity by restoring PPARγ expression, highlighting miR-130a as a potential therapeutic target for cardioprotection [65].

Collectively, miR-130a appears to play a key role in the pathogenesis of anthracycline-induced cardiotoxicity. However, further studies are required to validate its potential as an early biomarker and therapeutic target for cardioprotection.

## 4. Alarmins (DAMPs): Concept and Biological Functions

### 4.1. Definition and Main Characteristics

The immune system responds to exogenous damage signals following receptor engagement with pathogen-associated molecular patterns (PAMPs). Nevertheless, immunostimulation is also provoked by endogenous molecules termed damage-associated molecular patterns (DAMPs) upon cellular stress or death induced by physical, chemical, metabolic, or infectious insults [8].

The existence of DAMPs was first postulated in 1994 by Polly Matzinger, who proposed the “Danger Theory”, according to which the immune system is activated by intracellular molecules released by injured tissue. In other words, danger signals are not only exogenous but also endogenous, thus explaining immune responses elicited in sterile conditions [193].

Under physiological conditions, DAMPs do not stimulate the immune system, but they alert cells and induce an inflammatory response in the presence of cell damage. More precisely, DAMPs represent intrinsic danger signal molecules that are released from damaged, stressed, or necrotic cells, which then bind, as PAMPs, to pattern recognition receptors (PRRs), thereby initiating immune responses and inflammatory signaling cascades that serve essential regulatory functions in various pathophysiological phenomena [194,195].

Currently, different intracellular DAMPs have been identified. Specifically, they are divided into five classes: nucleic acids, proteins, ions, glycans, and metabolites [196]. The archetype DAMP is high mobility group box 1 (HMGB1) [197], a protein that is not only highly abundant but also evolutionarily conserved, present in nearly all eukaryotic cells [198], and has been shown to possess numerous significant biological functions both intracellularly and extracellularly.

Cells can sense not only DAMPs that are located within the cell itself but also DAMPs that originate from neighboring cells or even from distant tissues or organs [199]. Intracellularly, they can be present in the nucleus or cytoplasm, where corresponding DAMP-sensing receptors sense them; intercellularly, they can be sensed by both membrane and intracellular receptors [196].

DAMP-sensing receptors are different and include TLRs, C-type lectin receptors (CLRs), nucleotide-binding oligomerization domain-like receptors (NLRs), RIG-I-like receptors (RLRs), receptor for advanced glycation endproducts (RAGE), triggering receptor expressed on myeloid cells (TREM)1, TREM2, and soluble pattern recognition molecules such as pentraxins, collectins and ficolins [200,201]. While all DAMP-sensing receptors contribute to the detection of cellular damage and the initiation of inflammation, they differ in their specific targets, cellular locations, and the precise molecular cascades they trigger, leading to varied physiological and pathological outcomes [200]. For example, TLRs often activate the NFκB and MAPK pathways, leading to the secretion of inflammatory cytokines and chemokines [202], while RAGE activates transcription factors like NFκB, AP-1, and STAT3 [203].

### 4.2. Mechanisms of Release and Signaling

Alarmins can be released through different mechanisms: passive release or active secretion. Passive release is the result of different types of cell death, i.e., apoptosis, necroptosis, pyroptosis, ferroptosis, or cuproptosis [204]. During cell death, there is a loss of plasma membrane integrity. Interestingly, although the integrity of the plasma membrane is maintained in apoptotic cells, apoptosis induced by chemotherapy or other therapies can promote the release of certain DAMPs by different mechanisms, including nucleosomal DNA fragmentation, *via* the plasma membrane channel pannexin 1 (PANX1) or through microparticles [205,206,207,208].

Alternatively, DAMPs can be released by active secretion via lysosomes and exosomes. Both lysosomes and exosomes carry different DAMPs, and once they reach the plasma membrane, they are discharged via the RAB–SNARE complex [209,210].

Once in the extracellular space, DAMPs exert their biological effects through their receptors, activating complex signaling pathways that can lead to tissue repair and immune activation. Nevertheless, when their release is chronic or excessive, it can contribute to inflammatory conditions [200]. In the context of cancer and CVDs, DAMPs also affect the balance among inflammation, immunity, and tissue repair [195]. Specifically, they are released from dying tumor cells and induce inflammation, thereby facilitating tumor growth. Furthermore, they enhance tumor cell metastasis by promoting cell migration [211]. Nevertheless, DAMPs also mediate antitumor effects (i.e., tumor-specific immune responses) once released following the immunogenic cell death of cancer cells induced by certain chemotherapeutic drugs [212].

The following DAMPs have been related to cardiotoxicity induced by antitumor treatments and are summarized in Table 3.

## 5. Alarmins Involved in Cardiotoxicity, Modulated by Cancer Therapies of Skin and Breast Cancers

### 5.1. HMGB1

High-mobility group box 1 (HMGB1) is a multifunctional nuclear protein that, upon cellular stress or death, can be released extracellularly and act as a DAMP [228]. In breast cancer, extracellular HMGB1 has a dual role, contributing to tumor-promoting inflammation, immune evasion, and tumor progression [229]. In melanoma, HMGB1/RAGE and HMGB1/TLR4 signaling have been implicated in tumor growth, angiogenesis, metastasis, and immune suppression through the accumulation of IL-10-producing M2 macrophages and the modulation of IL-23/IL-17 pathways [230]. Thus, HMGB1 represents a context-dependent molecular link between cellular stress, inflammation, and tumor progression.

In the infarcted heart, extracellular HMGB1 first recruits monocytes to the site of injury, which then secrete inflammatory cytokines, thereby driving an inflammatory response. Notably, HMGB1 also supports cardiac tissue repair by modulating macrophage polarization toward a tissue-healing phenotype and suppressing dendritic cells (DCs), cytokine secretion and accumulation. Furthermore, HMGB1 has been shown to contribute to cardiac repair and regeneration by activating stem cells, endothelial cells, and endothelial progenitor cells, and by modulating fibroblasts [231].

Following MI, HMGB1 directly modulates the activity of different immune cell types to orchestrate an inflammatory reparative response (IRR). MI is characterized by an initial acute inflammatory response triggered by the innate immune system. Specifically, cell necrosis and ECM degradation generate DAMPs, such as HMGB1, that bind neutrophils and macrophages, which are fundamental to removing necrotic cell debris from the infarcted area. The early inflammatory phase is progressively replaced by the proliferative/reparative phase, driven by anti-inflammatory monocytes/macrophages that promote heart repair, enabling wound healing and proper scar formation. Finally, a maturation phase associated with ECM remodeling lasts several months [232]. In addition to its influence on cardiac recovery through the direct modulation of immune cells, HMGB1 has been shown to play a significant role in cardiac repair and regeneration by stimulating stem and progenitor cells and affecting fibroblasts [228]. HMGB1 exerts its pleiotropic functions depending on the oxidation status of its cysteine residues [i.e., reduced state (fr-HMGB1), disulfide form (ds-HMGB1), oxidized state (ox-HMGB1)] [233]. These different forms bind to different receptors and exert different biological functions. Following MI, HMGB1 is released in its reduced form, and in the oxidizing microenvironment generated by ROS, is first converted to the disulfide form and later to the oxidized form. Fr-HMGB1 recruits inflammatory cells and cardiac fibroblasts, promoting tissue regeneration by inducing macrophage polarization. Ds-HMGB1 stimulates the release of inflammatory and angiogenic factors and can be further oxidized to sulfonyl ox-HMGB1, which is mainly associated with the resolution of inflammation. It is noteworthy that although ox-HMGB1 is irreversible, fr-HMGB1 and ds-HMGB1 can interconvert [233].

Several chemotherapeutic drugs exert long-term cardiovascular complications mainly by leading to the upregulation of several pro-inflammatory and oxidative stress markers, including HMGB1 [234]. AC and platinum-based agents are classic examples of cardiotoxic drugs. Specifically, adriamycin (ADR) is one of the most effective AC antitumor agents in the treatment of specific hematological malignancies and solid tumors. Nevertheless, its cardiotoxicity limits its clinical use. Specifically, ADR induces cardiomyocyte autophagy, followed by cardiac damage, and HMGB1 contributes to these effects, as demonstrated by the decreased expression of apoptotic markers and mitochondrial membrane potential following HMGB1 silencing in an *in vivo* rodent model of ADR-induced cardiotoxicity [213].

DOX is another anthracycline widely used as an antitumor agent. The clinical application of this drug is also limited due to its capacity to induce mitochondrial impairment, ROS generation, and autophagic dysregulation, leading to cardiomyopathy [235]. Recently, it has been demonstrated that ferroptosis may be the main cause of DOX-induced cardiomyopathy in rats, and that HMGB1 appears to be a downstream regulator of this newly defined programmed cell death process [214]. Interestingly, HMGB1 mediates DOX-induced ferroptosis through neutrophil extracellular traps (NETs). Mechanistically, HMGB1, once triggered by NET formation, mediates this type of cell death in cardiomyocytes by binding to and activating TLR4, thereby modulating YAP and activating the Hippo pathway as demonstrated in a murine model of DOX-induced cardiotoxicity [215]. A very recent study, using mouse models and human organotypic myocardial slices, demonstrated that CCDC25, a transmembrane DNA receptor on cancer cells that senses NET DNA and promotes cancer metastasis, contributes to NET-mediated cardiotoxicity. Specifically, CCDC25 is expressed on the cardiomyocyte cytomembrane, and the NET–CCDC25 interaction promotes ROS generation and modulates autophagic flux, thereby inducing cardiotoxicity [236].

More recently, other agents, such as tyrosine kinase inhibitors (TKIs), have also been involved in adverse cardiovascular outcomes [237]. Imatinib (Imtb)—the first FDA-approved TKI—represents one of these agents. Specifically, treatment with Imtb leads to the upregulation of several pro-inflammatory and oxidative stress markers in cardiac tissue, including HMGB1 [216].

RT plays an essential role in the treatment of more than 70% of all cancer types. However, it also exerts side effects, with radiation-induced heart disease (RIHD) representing one of the main causes of noncancer-related death [238]. RIHD consists of accelerated atherosclerosis, adverse myocardial remodeling, conduction abnormalities, fibrosis, and acute pneumonitis risk, all conditions that lead to heart failure several years after exposure to radiation therapy [239,240]. Specifically, RT induces the excessive generation of ROS and reactive nitrogen species, followed by apoptotic cell death and the release of DAMPs. Interestingly, several clinical and preclinical studies have shown a critical role for the innate immune receptor TLR4 in RIHD [241,242], and HMGB1 binds directly to this receptor, thereby activating pro-inflammatory signaling pathways, including the NF-κB pathway [243].

Recently, several studies have shown evidence of RT’s synergistic effect with other anticancer therapies, such as immunotherapy [244]. Nevertheless, this combination can increase the superimposed toxic side effects, such as cardiotoxicity. For instance, when radiotherapy is combined with programmed death 1 (PD-1) inhibitors in tumor-bearing mice, there is an exacerbation of myocardial injury because radiation induces passive release of HMGB1 from necrotic cardiomyocytes, and PD-1 inhibitors synergistically induce HMGB1, thereby aggravating the inflammatory response and myocardial damage [217,218,245].

### 5.2. Nucleophosmin

Nucleophosmin (NPM, also known as NPM1, B23, Numatrin, and nucleolar protein NO38) is a multifunctional phosphoprotein shuttling between the nucleolus and cytoplasm [246,247] involved in the transport of proteins, in fact, it belongs to the nucleoplasmin/nucleophosmin family of nuclear chaperones [248,249]. Intracellular NPM has different roles, including DNA damage repair and the cell cycle [250,251]. It regulates the stability and activity of transcription factors, including p53 and nuclear factor kappa B (NFκB) [252,253].

Moreover, NPM acts as a stress sensor in endothelial cells being secreted under stress conditions. It promotes angiogenesis and inflammation, contributing to vascular disease and endothelial dysfunction through mechanisms that involve the NF-κB pathway [254].

NPM has been widely studied in the tumor context since it plays an important role; in particular, mutations and dysregulation of NPM play a major role in cancer development and progression. In acute myeloid leukemia (AML), heterozygous NPM1 mutations (NPM1c+) are among the most common genetic alterations and generate a truncated protein that aberrantly localizes to the cytoplasm, promoting leukemogenesis [255] In solid tumors, NPM is overexpressed, including in triple-negative breast cancer (TNBC), an aggressive, fast-growing subtype characterized by cells lacking estrogen (ER), progesterone (PR), and HER2 receptors; therefore, TNBC does not respond to hormonal therapy or HER2-targeted drugs. Indeed, knockdown of NPM has been exploited as a therapeutic strategy to impair the proliferative capacity of TNBC cells [256].

Also in skin cancers such as melanoma [257], gastric, lung, and liver cancers, NPM is frequently overexpressed and is associated with increased proliferation, high metastatic potential, and poor prognosis [258]. Moreover, elevated NPM expression contributes to chemoresistance by helping tumor cells evade apoptosis and survive anticancer treatments [258].

Recently, NPM has also been described to act as an alarmin. NPM has been detected in the extracellular environment upon lipopolysaccharide (LPS) stimulation from human macrophages. Extracellular NPM, in turn, triggers inflammatory signaling pathways, supporting its role as an alarmin-like molecule released by damaged or stressed tissues to activate immune and inflammatory responses [259].

Our study supports this concept and demonstrates in detail the role of NPM as an alarmin, showing that it is actively released by human cardiac progenitor cells before the onset of cytotoxicity following ultraviolet radiation or DOX exposure through a non-canonical autophagic mechanism involving TLR4-dependent secretion [9]. Moreover, NPM is also upregulated in the plasma of a DOX-induced cardiotoxicity mouse model [9]. The secreted NPM further stimulates TLR4 signaling, creating an inflammatory loop that affects cardiac repair, and at least in part, explains the cardiotoxic effect of DOX [9]. Thus, the release of NPM upon DOX treatment is one of the mechanisms involved in cardiac damage.

Its role in cardiotoxicity has also been investigated in a separate study of psoriasis, an inflammatory disease associated with increased CVD risk [260,261,262]. NPM has been shown to increase in the cutaneous compartment, to be released by both cutaneous fibroblasts and keratinocytes in response to inflammatory stimuli, and to be upregulated in the plasma of psoriatic patients compared with healthy controls [263,264]. Moreover, circulating NPM positively correlates with the severity of disease assessed by the psoriasis area severity index (PASI) and with determinants of CVDs such as left ventricular mass, systolic pressure, and pulse wave velocity an index of arterial stiffness [263].

Taken together, NPM represents a molecular bridge between cancer biology and cardiovascular inflammation, with potential implications as a biomarker and therapeutic target for both malignancies and cancer therapy-associated cardiotoxicity.

### 5.3. Heat Shock Proteins

Heat shock proteins (HSPs) are intracellular molecular chaperones that protect cells from stress by assisting protein folding, preventing protein aggregation, and regulating apoptosis. More recently, HSPs have also been identified in the extracellular environment, where they behave similarly to DAMPs, activating immune and inflammatory responses through receptors such as TLR2 and TLR4 [265].

Extracellular HSPs are elevated in different cancers, including breast cancer, where they support tumor survival, metastasis, inflammation, and resistance to therapy [266].

In melanoma, HSPs, particularly HSP70, HSP90, and HSP27, promote melanoma progression by stabilizing oncogenic proteins, enhancing tumor cell survival, supporting metastasis, and helping cancer cells evade apoptosis and immune surveillance. Their overexpression is associated with aggressive disease and has made HSPs attractive therapeutic targets in melanoma [267,268,269].

In CVDs, it has been shown that circulating HSP60 and HSP70 levels correlate with disease severity and prognosis in HF. Indeed, misfolded proteins accumulate during cardiac stress, hypertrophy, and ischemia, and HSPs initially act protectively by restoring protein homeostasis. Chronic or dysregulated extracellular HSP signaling may instead promote inflammation, apoptosis, fibrosis, and cardiac dysfunction [270]. Interestingly, HSPs are induced by oxidative stress, smoking, hypercholesterolemia, and vascular injury. In fact, extracellular HSP60 and HSP70 activate immune cells and cytokine production, contributing to vascular inflammation and plaque development [271]. Anti-HSP antibodies correlate with the severity of atherosclerosis, suggesting diagnostic potential. Additionally, HSP70 and HSP90 increase after ischemic stress and appear cardioprotective [272]. They have been shown to reduce oxidative damage, improve resistance to hypoxia, and may limit infarct size. Interestingly, ischemic preconditioning and exercise can induce HSP expression [273].

Finally, HSP90 contributes to angiotensin II signaling, vascular smooth muscle proliferation, endothelial dysfunction, and pulmonary hypertension. HSP70 may exert opposite protective effects against hypertensive remodeling [274].

Importantly, HSPs are implicated in chemotherapy-associated cardiotoxicity; they play a dual role, acting both as protective chaperones and as mediators of damage when dysregulated.

Various HSPs are upregulated in cardiac tissues of different animal models following DOX administration, including HSP47, HSP60, HSP10, and HSP90. Some of them contribute to long-term cardiac dysfunction; for example, HSP47 promotes the NLRP3 inflammasome, causing cardiac atrophy and fibrosis in mice [219], on the contrary, HSP60 and HSP10 are involved in post-translational modifications of Bcl-2 proteins under DOX stress, reducing apoptosis in rat cardiomyocytes [220], and HSP90 protects cardiomyocytes to survive to DOX-induced stress stabilizing protective proteins such as the receptor tyrosine-protein kinase erbB-2 (ErbB2) survival signaling in rats [222], and PI3K/Akt and IKK2 in mouse hearts [221].

Finally, DOX treatment downregulates HSP20, which plays a protective role by reducing DOX-associated oxidative stress and improving AKT phosphorylation; indeed, the overexpression of HSP20 in cardiomyocytes confers greater resistance to DOX-induced cell death. HSP20, in fact, interacts with p-AKT, protecting it against dephosphorylation by protein phosphatase 1 (PP1) [223].

HSPs, therefore, may become biomarkers for HF and cardiovascular stress. Pharmacological modulation of HSPs or the inhibition of extracellular HSP signaling could represent future therapies.

### 5.4. S100 Proteins

The S100 protein family is a small acidic protein with a structural core characteristic of S100 proteins, highly conserved, defined by their common ability to dissolve in 100% ammonium sulfate [275]. They have two distinct conserved EF-hand domains, which are small structural motifs that bind calcium ions (Ca^2+^). These functional domains are key calcium-binding regulators involved in cardiac homeostasis and cardiac disease progression. Different S100 members have context-dependent roles; for example, S100A1 supports cardiomyocyte contractility, calcium handling, and mitochondrial function. After myocardial ischemia, cardiomyocytes and dying cells release S100A1 into the blood [276].

S100 proteins, particularly S100B, are highly expressed in melanoma and promote tumor progression by inhibiting the tumor suppressor p53 and enhancing cell proliferation, survival, invasion, and metastatic potential. Elevated S100B levels are associated with advanced disease stage and poor prognosis, making it both a clinically useful biomarker and a potential therapeutic target in melanoma [277].

In breast cancer, members of the S100 family, especially S100A4, S100A7 (psoriasin, a molecule associated with psoriasiform hyperplasia), and S100A8/A9 (calprotectin), contribute to tumor growth, invasion, metastasis, and therapy resistance by regulating inflammatory and pro-survival signaling pathways. Increased expression of these proteins is frequently associated with aggressive tumor phenotypes, poor clinical outcomes, and enhanced metastatic dissemination [278,279,280].

S100 proteins also play a major role in CVDs. After myocardial ischemia, cardiomyocytes and dying cells release S100A1 into the blood [276]; therefore, circulating S100A1 levels can serve as a biomarker for acute myocardial ischemia [281]. Other S100 proteins, i.e. S100A8/A9 and S100B, instead act as pro-inflammatory alarmins that promote oxidative stress, endothelial dysfunction, and adverse cardiac remodeling [282,283]. Therefore, S100 proteins are both biomarkers and mediators of cardiovascular injury, with potential diagnostic and therapeutic relevance in HF, ischemic injury, and inflammatory CVD. Since cardiomyopathy occurs in type 2 diabetic patients, as well as in cancer patients undergoing DOX chemotherapy, a study evaluated the effects of DOX in db/db mice and identified the molecular pathways responsible for DOX-induced cardiotoxicity in type 2 diabetic hearts [224]. Interestingly, S100A8/A9 are upregulated by DOX treatment in diabetic mice. Their expression pattern correlates with worsening cardiac performance, suggesting a mechanistic link. Their upregulation induced NF-κB signaling and activation of the p38 MAPK pathway, leading to higher levels of inflammatory cytokines in the myocardium. The authors conclude that S100A8 and S100A9 are key inflammatory mediators that link diabetes with increased susceptibility to DOX-induced cardiac damage and may represent potential therapeutic targets or biomarkers for cardiotoxicity in diabetic cancer patients [224].

In DOX-induced cardiac toxicity, S100A1 levels are often reduced, leading to impaired sarcoplasmic reticulum Ca^2+^ cycling, reduced contractile performance, and increased susceptibility to apoptosis, contributing to overall contractile failure. Indeed, several treatments used as cardioprotective agents against DOX-induced cardiomyopathy, such as the β-blocker carvedilol (CAR) and the natural polyphenol resveratrol, reduce DOX-induced cardiotoxicity in rats and improve cardiac function. This protective effect has been associated with the decrease in inflammation and oxidative stress, as well as an increase in S100A1 levels [225].

Collectively, these findings highlight the dual role of S100 proteins as both regulators of cardiac function and mediators of inflammation, linking cancer, CVDs, and therapy-induced cardiotoxicity. Their use as biomarkers and therapeutic targets could contribute to the early detection and mitigation of cardiovascular complications in cancer patients.

### 5.5. IL-33

IL-33 is an alarmin, identified in 2005, belonging to the IL-1 family of cytokines and a T-helper type 2-associated immune response inducer [284] that is expressed in in different cells including cardiomyocytes, fibroblasts, endothelium, and epithelium [285]. It is a multifunctional cytokine that plays different roles in pathological conditions, such as CVDs, allergy, infectious diseases, and cancer, by acting on multiple types of immune cells and promoting type 1 and 2 immune responses, playing roles in maintaining host homeostasis [286].

IL-33 is released by immune and non-immune cells upon stress, acting as an “alarmin”, binding suppression of tumorigenicity 2 (ST2 receptor, also called IL-33R or IL-1R4) and its co-receptor IL-1 receptor accessory protein (IL-1RacP, also known as IL-1R3), activating inflammatory and immune response pathways [287].

In melanoma, IL-33 can act as a pro-tumorigenic cytokine by enhancing tumor growth, invasion, and immune evasion through activation of the ST2 receptor pathway. However, in some contexts, it may also promote antitumor immune responses depending on the tumor microenvironment [288].

In breast cancer, IL-33 is generally associated with tumor progression by promoting proliferation, metastasis, angiogenesis, and immunosuppression, largely through the recruitment of myeloid cells and skewing toward a pro-tumoral inflammatory milieu *via* the IL-33/ST2 axis [289].

It was reported that different pro-inflammatory cytokines such as tumor necrosis factor (TNF)-α, interferon (IFN)-γ, and IL-1β significantly increased both the protein and mRNA levels of IL-33 in primary, human adult cardiac myocytes, cardiac fibroblasts, vascular endothelial cells, coronary artery smooth muscle cells, and macrovascular (aortic and coronary artery) and heart microvascular endothelial cells in vitro [290].

It plays different roles in CVDs; for example, in viral cardiac injuries, IL-33 inhibits inflammation by inducing a Th2-type immune response and M2 macrophage polarization. In contrast, in the chronic phase, IL-33 induces eosinophil infiltration. It increases inflammatory cytokine levels, resulting in dilated cardiomyopathy [291]. In CAD, IL-33 is induced and plays a protective role. In fact, it causes ERK1/2 phosphorylation and IL-10 and ATP-binding cassette transporter A1 (ABCA1) upregulation; therefore, it is responsible for HDL formation and reverse cholesterol transport, thus preventing foam cell formation [292,293].

In CAD, serum sST2 concentrations were increased appreciably in patients with ST-segment elevation MI and to predict mortality in the cohort [294]. sST2, which lacks the transmembrane domain, is secreted into the extracellular space and serves as a decoy receptor that binds IL-33. Thus, increased circulating levels of IL-33 and sST2 are associated with adverse outcomes in CAD.

After MI, both sST2 and IL-33 levels are increased compared to normal healthy controls, with a further increase in patients with HF [295]. In MI, the IL-33/ST2L axis plays a dual role, being cardioprotective in the acute phase by suppressing inflammation and promoting repair, while in the chronic phase is detrimental, enhancing Th2-driven inflammation, fibrosis, and adverse cardiac remodeling [285].

Experimental studies demonstrate that IL-33/ST2L signaling protects against pressure overload-induced cardiac hypertrophy and fibrosis. In contrast, elevated sST2 antagonizes these beneficial effects by blocking IL-33 binding to ST2L, thereby exacerbating cardiac injury [296].

In diabetic cardiomyopathy (DCM), IL-33 is generally downregulated. Its supplementation or overexpression reduces cardiomyocyte apoptosis by limiting ER stress, lipid accumulation, and proapoptotic signaling pathways (including PKCβII/DGK-ζ modulation), thereby improving cardiac function and diastolic performance [297]. Conversely, increased circulating sST2 in diabetic patients correlates with worse diastolic dysfunction and adverse cardiovascular outcomes, supporting the concept that impaired IL-33/ST2L signaling contributes to myocardial injury. In contrast, higher sST2 reflects disease severity and reduced cardioprotective IL-33 activity [298]. Interestingly, metformin has been shown to improve cardiac remodeling after MI by increasing IL-33 expression and reducing sST2 levels [299].

IL-33 has been reported to exert cardioprotective and disease-modulating effects in several cardiac conditions, including rheumatic heart disease, atrial fibrillation, acute HF, and acute aortic syndrome, often through pathways involving TGF-β-mediated fibrosis and inflammatory regulation. In many of these diseases, circulating IL-33, and especially sST2 levels, are elevated and correlate with disease severity, such as in atrial fibrillation and idiopathic inflammatory myopathies [285]. Overall, the IL-33/ST2 axis appears to reflect both cardiac involvement and remodeling activity, with increased sST2 consistently associated with worse clinical status and potentially indicating impaired IL-33-mediated protective signaling.

In this scenario, anticancer drugs targeting IL33/ST2 modulate both tumor and cardiac functions.

In breast cancer patients receiving AC-containing chemotherapy, early increases in soluble ST2 (sST2) and NT-proBNP are associated with a higher risk of developing cardiac arrhythmias during treatment, suggesting early myocardial stress and injury [226]. Similarly, in breast cancer patients undergoing thoracic RT, it has been found that radiation exposure is associated with increased circulating sST2 levels, reflecting early cardiac stress and injury [227]. Therefore, dysregulation of the IL-33/ST2 pathway—particularly elevated sST2 as a decoy receptor—may contribute to radiation-induced cardiac damage, supporting its potential role as an early biomarker of cardiotoxicity.

## 6. Intersection Between microRNAs and Alarmins

**Figure 2:**
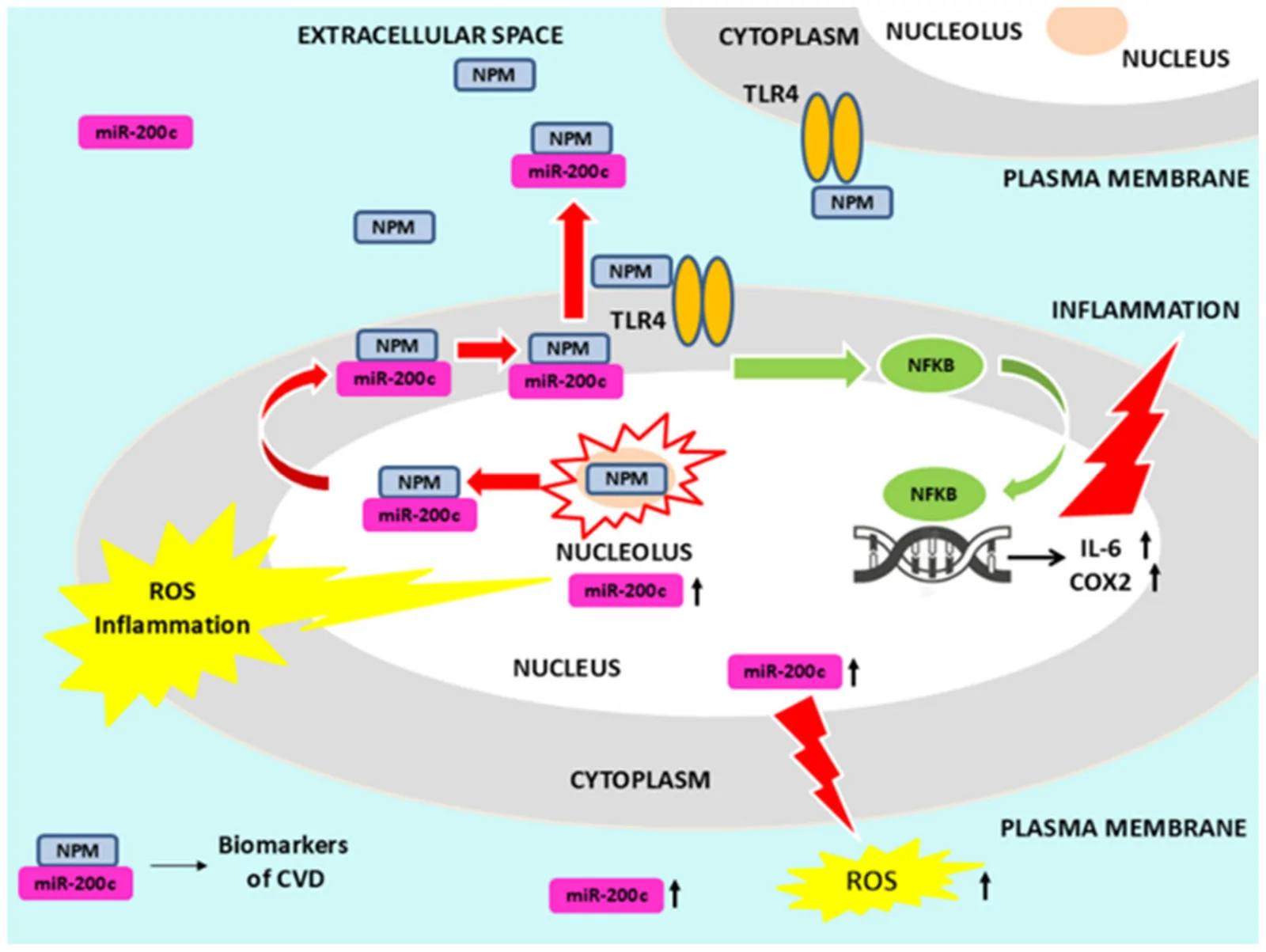
Interaction between NPM alarmin and miR-200c regulates ROS and inflammation. ROS and inflammation cause nucleolar stress eliciting NPM translocation from the nucleolus to the nucleus, and then to the extracellular space. NPM is an alarmin able to bind and protect miR-200c from RNase degradation. Thus, NPM might be responsible for miR-200c extracellular export and protection upon cytokine MIX exposure or cellular stress exposure *via* a TLR4 mechanism. This mechanism has been described in a chronic inflammatory diseases, such as psoriasis, where NPM and miR-200c are increased in both the skin and plasma. Therefore, NPM and miR-200c could be considered as novel biomarkers and potential targets to counteract chronic inflammation associated with CVD risk. Given the central role of oxidative stress and inflammation in CTRCD, it is plausible that a similar NPM–miR-200c regulatory axis may contribute to cardiac injury in the cardio-oncology setting.

Although further studies are needed to establish a direct link between alarmins and miRNAs, a strict link has already been demonstrated. Indeed, NPM displays a nucleic acid-binding domain [232] and a fraction of miRNAs, actively released in the extracellular space by cancer cells upon serum deprivation, are bound and protected from degradation by NPM [293]. Moreover, NPM can bind to circulating miR-200c in the plasma of patients with psoriasis and in the supernatants of skin cells exposed to inflammatory stimuli, demonstrating an intersection between alarmin and microRNA active release upon inflammatory stimuli through a mechanism that requires TLR4 [246] (Figure 2). Given the central role of oxidative stress and inflammation in CTRCD, it is plausible that a similar NPM–miR-200c regulatory axis may contribute to cardiac injury in the cardio-oncology setting.

## 7. Conclusions

This review highlights the dual role of miRNAs and alarmins in cancer therapy-induced cardiovascular toxicity, emphasizing their potential as circulating biomarkers for early detection, monitoring, and risk stratification. Therapeutic strategies targeting this axis—including miRNA mimics/inhibitors and alarmin modulators/inhibitors—may provide synergistic benefits by reducing cardiotoxicity while suppressing tumor growth. Each of these therapeutic strategies should be carefully studied, since they can impact cancer progression, but conversely could worsen cardiac stress and *vice versa*.

Therefore, a therapeutic approach that considers the possible interplay between alarmins and miRNAs should be carefully evaluated, and future studies should evaluate the potential physical and molecular interactions between these novel biomarkers.

## Figures and Tables

**Table 2:** miRNA modulated by anticancer treatments.

miRNA	Cancer Treatment	Modulation	Tissue/Cells	Source	Reference
miR-200c	DOX	Up	hCmPC	Human	[38]
	DOX	Up	LV heart	Mouse	[38]
miR-200a	DOX	Down	Rat cardiomyocytes	Rat	[39]
miR-200b	DOX	Up	Heart	Mice	[40]
let-7f	Epirubucin	Down	Blood	Human	[41]
let-7g	DOX	Down	Heart	Rat	[42]
miR-34a	DOX	Up	Myocardium, plasma	Rat	[43]
	anti-PD-1 inhibitor	Up	Cardiac macrophages	Mouse	[44]
miR-34b/c	DOX	Up	Cardiomyocyte cell line	Mouse	[45]
miR-29 family	AC	Up	Plasma	Young cancer pts	[46]
	DOX	Down	Myocardium, Cardiomyocytes	Rat	[47]
	IR	Down	Arteries	Human	[48]
miR-30 family	DOX	Down	Cardiomyocytes and heart	Rat	[49]
miR-140 5p	DOX	Up	Cardiomyocytes	Rat	[50]
miR-144	DOX	Down	Cardiomyocytes	Rat	[51]
miR-146a	DOX	Up	Cardiomyocytes	Human	[52,53]
	DOX	Up	Cardiomyocytes	Mouse	[53]
miR-21	DOX	Up	Myocardium	Mouse	[54]
	IR	Up	Myocardium	Rat	[55]
miR-210	AC	Down	Plasma	Breast cancer pts	[41]
miR-1	DOX	Up	Plasma	Breast cancer pts	[56,57]
	IR	Down	Myocardium	Rat	[58]
miR-133a	DOX	Up	Serum	Breast cancer pts	[59]
miR-208a	DOX	Up	Myocardium	Mice	[60]
	DOX	Up	Plasma	Rat	[61]
	DOX	Down	Myocardium	Rat	[40]
miR-208b	DOX	Up	Myocardium	Rat	[61]
	DOX	Up	Myocardium	Mouse	[40]
miR-499	AC	Up	Plasma	Breast cancer pts	[62]
	DOX	Down	Cardiomyocytes, heart	Mouse	[63]
	DOX	Up	Serum	Mouse	[63]
miR-126	AC	Down	Plasma	Breast cancer pts	[41]
	IR	Up	Endothelial extracellular vesicles	Mouse	[64]
miR-130a	DOX	Up	Cardiomyocytes	Mouse	[65]

Abbreviations: AC, anthracycline chemotherapy; DOX, doxorubicin; IR, ionizing radiation; pts, patients.

**Table 3:** Alarmins modulated by anticancer treatments.

Alarmin	Cancer Treatment	Modulation	Tissue/Cells	Source	Function	Reference
HMGB1	Adriamycin	Up	Heart, cardiomyocytes	Mice	Cardiomyocyte damage by autophagy	[213]
	DOX	Up	Heart, cardiomyocytes	Mice	Cardiomyocyte damage by ferroptosis	[214]
	DOX	Up	Heart, cardiomyocytes	Mice	Cardiomyocyte damage by ferroptosis	[215]
	Imatinib	Up	Heart	Rat	Inflammation	[216]
	RT + PD-1 inhibitors	Up	Heart	Mice	Inflammation	[217]
	RT + PD-1 inhibitors	Up	Heart	Mice	Cardiomyocyte damage by pyroptosis	[218]
NPM	DOX	Up	Supernatants of hCMPCs	Human	Inflammation	[9]
	DOX	Up	Plasma	Mice	Inflammation	[9]
HSP 47	DOX	Up	Cardiomyocytes, serum, heart	Mice	Inflammation, Cardiac atrophy and fibrosis	[219]
HSP 60	DOX	Up	Cardiomyocytes	Rat	Antiapoptotic	[220]
HSP 10	DOX	Up	Cardiomyocytes	Rat	Antiapoptotic	[220]
HSP 90	DOX	Up	Cardiomyocytes, heart	Chicken, mouse	Antiapoptotic, Oxidative stress Reduction	[221]
	DOX	Up	Heart, cardiomyocytes	Rat	Increased viability, Cardioprotection	[222]
HSP 20	DOX	Down	Cardiomyocytes	Mice	Apoptotic, oxidative stress increase	[223]
S100A8/A9	DOX	Up	Heart	Diabetic mice	Inflammation	[224]
S100A1	DOX	Down	Heart	Rats	contractile failure, inflammation, Oxidative stress Increase	[225]
sST2	AC	Up	Blood	Breast cancer pts	Il33 binding, Increased Cardiac arrhythmia	[226]
	RT	Up	Serum	Breast cancer pts	Myocardial damage	[227]
IL33/sST2	X-ray	Up	Heart	Mice	Cardiac dysfunction	[227]
	X-ray	Up	Cardiomyocytes	Rat	Cell injury	[227]

Abbreviations: AC, anthracycline chemotherapy; DOX, doxorubicin; IR, ionizing radiation; pts, patients; RT, radiotherapy.

## Data Availability

No new data were created or analyzed in this study. Data sharing is not applicable to this article.
